# Supplementary material for: Designing intervention prototypes to improve infant and young child nutrition in Peru: a participatory design study protocol
Source: BMJ Open. 2023 Dec 8;13(12):e071280. doi: 10.1136/bmjopen-2022-071280 (PMC10729018; doi:10.1136/bmjopen-2022-071280)
Supplement: Supplementary data [file bmjopen-2022-071280supp001.pdf]

Designing intervention prototypes to improve infant and young child nutrition in Peru: A participatory design study protocol

E. K. Rousham, R. Pareja, H.M. Creed-Kanashiro, R. Bartolini, R. Pradeilles, D. Ortega-Roman, M. Holdsworth, P. Griffiths, N. Verdezoto

Supplementary materials

Supplementary Table 1: Summary of the 27 identified nutrition challenges, and 19 opportunities identified for infants and young children (IYC) aged 6-23 months in two peri-urban communities in Peru prior to the participatory co-design phase of the study

Overarching theme	Challenges identified in the formative research‡	Opportunity statements derived from the challenges
Improve the healthiness of maternal diets and infant & young child feeding practices	1. High consumption of sugar-sweetened beverages in mothers and IYC	1. Reduce the consumption of unhealthy foods and beverages amongst mothers and IYC to improve the overall quality of diet and feeding practices
	2. High consumption of unhealthy foods (savoury snacks/fried foods, sweet products) in mothers and IYC	
	3. Sub-optimal dietary diversity, particularly around animal-source foods and fruit and vegetable consumption, due to small quantities consumed	2. Improve the diversity of diets for mothers and IYC
	4. Inadequate engagement with responsive feeding in the home	3. Increase the engagement with responsive feeding practices at home
	5. Low prevalence of exclusive breastfeeding under six months, including early introduction of complementary foods†	4. Increase the prevalence of exclusive breastfeeding for infants under 6 months
	6. Low compliance with taking iron supplements in IYC†	5. Increase adherence to iron supplementation
	7. Issues with emotional and psychological wellbeing of mothers which impacts on the caring and feeding practices for the child	6. Improve emotional and psychological wellbeing for mothers to reduce its impact on IYC nutrition
	8. Mothers' understanding of nutrition knowledge and perception and practices around infant nutrition	7. Increase/enhance maternal nutrition knowledge on appropriate feeding practices (e.g., iron supplementation, complementary feeding, breastfeeding)
	6. Low compliance with taking iron supplements in IYC†	
	5. Low prevalence of exclusive breastfeeding under 6 months (which means that other liquids and complementary foods are introduced early)†	
	9. Challenges with regards to maternal time use in the home and outside the home	8. Improve the maternal care experience and practices at home/in everyday life -- e.g., tensions with work and time allocation
Enhance the local systems in the community to improve maternal and child nutrition	10. Lack of physical resources and materials	9. Enhance the physical and/or material resources within healthcare settings
	11. Issues with the organisation of consultations, including health centre structures and workflow	10. Optimise the organisation of consultations and workflow within healthcare settings
	12. Health service recommendations do not take into account the socio-cultural practices and norms of caregivers	11. Enhance the uptake, access, and intercultural approaches to improve maternal and child care services

Rousham E, *et al. BMJ Open* 2023; 13:e071280. doi: 10.1136/bmjopen-2022-071280

	13. Issues with the uptake and access to health services for IYC†	
	14. Issues with regards to counselling approaches and staff skills within health centres	12. Enhance counselling skills for healthcare professionals
	15. Community-based activities for breastfeeding outreach and support have national coverage	13. Facilitate community-based activities to enhance breastfeeding practices
	16. Inadequate counselling for complementary feeding	14. Improve nutrition counselling during pregnancy and from birth onwards
	17. Inadequate nutritional counselling for pregnant women	
	18. Restriction on infant formula promotion in ante-natal and post-natal settings	15. Promote the reduction of the use and promotion infant formula in antenatal and postnatal care settings
	19. Government policy should ensure food services in early childhood education provide and promote healthy food choices (e.g. via canteens, food at events, fundraising, promotions, vending machines)†	16. Help to implement governmental policies for early childhood education services to provide and promote healthy food choices
Support the national system to improve maternal and child nutrition policy implementation	20. Funding for interventions and policies to reduce obesity, stunting and iron deficiency anaemia in IYC	17. Seek funding opportunities to develop interventions and policies to address the double burden of malnutrition
	21. Monitoring systems particularly in vulnerable young children's populations for the five double duty actions and to track progress towards reducing nutritional inequalities	18. Develop strategies to reduce inequalities to protect vulnerable populations in relation to the double burden of malnutrition
	22. Reduce inequalities or protect vulnerable populations in relation to obesity, stunting and iron deficiency anaemia in IYC	
	23. Lack of use of ICT interventions for the double burden of malnutrition in low and middle income country settings*	
	13. Issues with the uptake and access to health services for IYC†	
	24. Restrict commercial influences on the development of policies related to preventing obesity, stunting and iron deficiency anaemia in IYC	
	25. Robust coordination to ensure alignment, and integration of policies in obesity, stunting and iron deficiency anaemia in IYC across departments and levels of government (national and local)	19. Address the challenges in coordinating the implementation and evaluation of policy across different levels of governmental institutions in relation to the double burden of malnutrition
	26. Lack of coherence in the policy environment (e.g. lack of a holistic nutrition approach, no attention to the double burden of malnutrition	
	19. Government policy should ensure food services in early childhood education provide and promote healthy food choices (e.g. via canteens, food at events, fundraising, promotions, vending machines)†	

	27. Low adherence to taking iron supplements, reasons include fear of side effects and difficulties of administering at home, and low quality iron supplements that government offers	

‡Shading represents the source of each challenge from Phase 1 work packages. Key: no fill = work package a) Dietary assessment and nutritional status of IYC and mothers; light grey = work package b) Caregiver drivers and household or family influences on IYC nutrition and complementary feeding; mid-grey = work package c) Health centre and day care centre influences on IYC nutrition and complementary feeding; dark-grey = work package d) Policy mapping and prioritisation of government-level actions to tackle multiple forms of malnutrition for IYC.

†Challenges that appeared under more than one overarching theme

*Challenge identified from reviewing literature

Rousham E, *et al. BMJ Open* 2023; 13:e071280. doi: 10.1136/bmjopen-2022-071280
