# Supplementary material for: Designing intervention prototypes to improve infant and young child nutrition in Peru: a participatory design study protocol
Source: BMJ Open. 2023 Dec 8;13(12):e071280. doi: 10.1136/bmjopen-2022-071280 (PMC10729018; doi:10.1136/bmjopen-2022-071280)
Supplement: Author's manuscript [file bmjopen-2022-071280.draft_revisions.pdf]

BMJ Open is committed to open peer review. As part of this commitment we make the peer review history of every article we publish publicly available.

When an article is published we post the peer reviewers' comments and the authors' responses online. We also post the versions of the paper that were used during peer review. These are the versions that the peer review comments apply to.

The versions of the paper that follow are the versions that were submitted during the peer review process. They are not the versions of record or the final published versions. They should not be cited or distributed as the published version of this manuscript.

BMJ Open is an open access journal and the full, final, typeset and author-corrected version of record of the manuscript is available on our site with no access controls, subscription charges or pay-per-view fees (http://bmjopen.bmj.com).

If you have any questions on BMJ Open's open peer review process please email info.bmjopen@bmj.com

BMJ Open

Designing intervention prototypes to improve infant and young child nutrition in Peru: A participatory design study protocol

Journal:	*BMJ Open*
Manuscript ID	bmjopen-2022-071280
Article Type:	Protocol
Date Submitted by the Author:	21-Dec-2022

Complete List of Authors:	Rousham, Emily; Loughborough University<br>Pareja, Rossina G. ; Instituto de Investigación Nutricional<br>Creed-Kanashiro, H.M.; Instituto de Investigación Nutricional<br>Bartolini, R.; Instituto de Investigación Nutricional<br>Pradeilles, Rebecca; Loughborough University<br>Ortega-Roman, Deysi; Cardiff University<br>Holdsworth, Michelle; IRD, NUTRIPASS<br>Griffiths, Paula; Loughborough University<br>Verdezoto, Nervo; Cardiff University

Keywords:	NUTRITION & DIETETICS, Community child health < PAEDIATRICS, PUBLIC HEALTH, Health policy < HEALTH SERVICES ADMINISTRATION & MANAGEMENT

SCHOLARONE™
Manuscripts

Designing intervention prototypes to improve infant and young child nutrition in Peru: A participatory design study protocol

E. K. Rousham[1*], R. Pareja[2*], H.M. Creed-Kanashiro[2], R. Bartolini[2], R. Pradeilles[1], D. Ortega-Roman[3], M. Holdsworth[4], P. Griffiths[1], N. Verdezoto[3]

*Authors contributed equally

Authors' affiliations

[1] Centre for Global Health and Human Development, School of Sport, Exercise and Health Sciences, Loughborough University, Loughborough, UK

[2] Instituto de Investigación Nutricional, Lima, Perú

[3] School of Computer Science and Informatics, Cardiff University, Cardiff, UK

[4] UMR MoISA (Montpellier Interdisciplinary Centre on Sustainable Agri-Food Systems), (Univ Montpellier, CIRAD, CIHEAM-IAMM, INRAE, Institut Agro, IRD), Montpellier, France

Corresponding authors

Prof E. K. Rousham, Centre for Global Health and Human Development, School of Sport, Exercise and Health Sciences, Loughborough University, Loughborough, LE11 3TU, UK. e.k.rousham@lboro.ac.uk

And

Dr N. Verdezoto, School of Computer Science and Informatics, Cardiff University, Cardiff, CF24 4AG, UK. verdezotodiasn@cardiff.ac.uk

For peer review only - http://bmjopen.bmj.com/site/about/guidelines.xhtml

Abstract

Introduction: Nutrition during the complementary feeding period (6-23 months) is critical to ensure optimal growth and reduce the risk of diet-related disease across the life course. Strategies to reduce multiple forms of malnutrition (stunting, overweight/obesity and anaemia) in infants and young children (IYC) are a key priority in low- and middle-income countries, including Peru. This study aims to co-design and develop prototypes for interventions to address the multiple forms of malnutrition in IYC in urban Peru, using a participatory design approach.

Methods and analysis: The study will be based within peri-urban communities in two areas of Peru (Lima and Huánuco city). Following the identification of key nutritional challenges for IYC aged 6-23 months through formative research (Phase 1), we will conduct a series of workshops bringing together healthcare professionals from government health centres and caregivers of IYC aged 6-23 months. Workshops (on idea generation; creating future scenarios; storyboarding, and early implementation and feedback) will take place in parallel in the two study areas. Throughout these workshops, we will engage with community participants to explore, experiment, co-design, and iteratively validate new design ideas to address the challenges around IYC complementary feeding from Phase 1. Workshop outputs and transcripts will be analysed qualitatively using affinity diagramming and thematic analyses. The intervention prototypes will be evaluated qualitatively and piloted with the participating communities.

Ethics and dissemination: Ethical approval for this study was obtained from the Ethical Review Committee of the Instituto de Investigación Nutricional (IIN) Peru (388-2019/CIEI-IIN), Loughborough University (C19-87) and confirmed by Cardiff University. Findings of the participatory design process will be disseminated through a deliberative workshop in Lima, Peru with national and regional government stakeholders, as well as participants and researchers involved in the design process. Further dissemination will take place through policy briefs, conferences, academic publications and illustrated booklets.

For peer review only - http://bmjopen.bmj.com/site/about/guidelines.xhtml

Strengths and limitations of this study

- Participatory co-design is an important way of incorporating the voice of the community in the design and development of health interventions.
- This participatory study will bring together healthcare providers and health service users in collaborative co-design workshops to promote active community involvement in infant and young child nutrition, and mutual learning for all participants including the researchers.
- The co-designed prototypes will be tailored to the nutritional challenges for infants and young children in peri-urban Peru.
- The experiences and ideas expressed by participants may be influenced by the impact of the COVID-19 pandemic on health services and access to healthcare in the previous two years.

For peer review only

For peer review only - http://bmjopen.bmj.com/site/about/guidelines.xhtml

Introduction

Nutrition during the complementary feeding period (6-23 months) is critical to ensure optimal growth and reduce the risk of diet-related diseases across the life course. In low-resource settings, the prevention of undernutrition in the form of stunting, wasting or micronutrient deficiencies has been the traditional focus of complementary feeding guidance (1–3). However, as populations go through dietary and nutrition transitions, infant and young child (IYC) diets are changing with increased consumption of sugar, fats and oils, refined grains and ultra-processed foods, bringing the additional risk of excess energy intake leading to overweight and obesity (4–7). This in turn leads to increased risk of diet-related diseases in later life. Strategies are now needed to address coexisting forms of malnutrition in infancy and childhood including stunting, micronutrient deficiencies and overweight/obesity (7–9).

Peru faces the public health challenge of multiple burdens of malnutrition with a high prevalence of iron-deficiency anaemia, stunting (low height-for-age) and a rapidly increasing prevalence of overweight and obesity (10). Peru has successfully reduced the rate of stunting in children under 5 years from 23.2% in 2010 to 12.1% in 2020 (10). This success has been attributed to anti-poverty policies, multi-sectoral government working and implementation of cross-cutting interventions in the poorest areas (11). In contrast, anaemia prevalence has changed little among children aged 6–36 months from 43.5% in 2015 to 40.0% in 2020 (INEI, 2015, 2021), despite this being a national priority since 2015. Since 2017, government strategies to address anaemia have focussed on national distribution of multi-micronutrient powders and iron supplementation for IYC through health services and promotion of iron-rich foods (12).

Additional concerns focus on the increasing prevalence of overweight and obesity in Peru, affecting 37.4% of children age 5-9 years (13). Cohort studies in Peru have shown rapid weight gain in infancy is associated with overweight and obesity at 8 years (14). Feeding practices in infancy or early childhood also shape future dietary behaviours, such that unhealthy complementary feeding practices may predispose to unhealthy diets in later life (6).

Although previous research has examined the influence of socio-cultural contexts on complementary feeding practices in relation to undernutrition (15), less attention has been given to exploring these influences on excess energy intake and overweight during early life

For peer review only - http://bmjopen.bmj.com/site/about/guidelines.xhtml

in Peru. Similarly, there are no integrated policies or interventions to prevent overweight alongside iron and micronutrient deficiencies in IYC.

Socio-technical approaches to healthcare technology design and implementation, such as human-centered design, co-design and community-based participatory research, have led to an increasing involvement of multiple stakeholders in the design process (16–18). Such methods can help multiple stakeholders, including community members, to express their thoughts, share ideas, experiment and actively participate (19) throughout the design process (20). However, most of these socio-technical initiatives have taken place in the Global North (21,22) highlighting the need for the development of new methods and tools to engage with Global South communities. While some previous studies have applied participatory co-design methods in Latin American or Peruvian contexts (23–27), few have involved technology in healthcare settings (28).

The aim of this study is to co-design and develop prototypes for interventions to address the multiple forms of malnutrition coexisting in IYC in Peru, using a participatory design approach. This co-design follows on from formative research in the communities and health services that used qualitative and quantitative data as well as policy mapping to assess the extent of malnutrition in peri-urban communities, underlying contributors to malnutrition, and the key nutritional challenges identified by researchers and community stakeholders.

Methods and analysis

Study background and Phase 1

The PERUSANO study is an interdisciplinary project which aims to address multiple forms of malnutrition among IYC aged 6-23 months in peri-urban communities in Peru. This protocol follows on from the first phase of research in the PERUSANO project. The formative research (Phase 1) aimed to identify the prevalence of all forms of malnutrition as well as the challenges and facilitators of healthy IYC feeding practices to inform the second phase of the project that will take a co-design approach with community stakeholders (healthcare professionals, mothers and family caregivers, and other community actors) in Peru to scope opportunities to address the identified IYC feeding challenges. The wider PERUSANO project team includes a cross-cultural and multidisciplinary group of researchers (seven from Peru and ten from UK/France) with backgrounds in public health, nutrition, social sciences, and human-computer interaction (HCI). Phase 1 objectives were achieved through four work packages: a) cross-sectional, quantitative assessment of dietary

For peer review only - http://bmjopen.bmj.com/site/about/guidelines.xhtml

intake and nutritional status (weight, length/height and haemoglobin concentration) of IYC and mothers alongside a household socio-demographic survey (n=244); b) and c) in-depth qualitative studies (semi-structured interviews, observations) of caregiver drivers of IYC feeding (e.g., feeding style, food attributes) within households (b) and during consultations at local health care centres (c), and d) policy mapping and prioritisation of government-level actions that exist to tackle multiple forms of malnutrition for IYC with sixteen national experts. An overview of the study design with Phase 1 work packages and Phase 2 components is shown in **Figure 1**.

Phase 1 started in December 2019 and was interrupted by the COVID-19 pandemic in March 2020. COVID-19 restrictions in Peru prevented face-to-face workshops or field work taking place from March 2020 to May 2022. During this time, we analysed data from Phase 1 and continued meetings with researchers and stakeholders via online platforms. An 11 month no-cost extension to the project was granted by funders due to the impact of COVID-19 which enabled face-to-face data collection to commence again in June 2022.

End of Phase 1: Identification of key infant and young child nutritional challenges for participatory co-design

In July 2021, we conducted an online scoping workshop with 19 members of the interdisciplinary team, where researchers presented the nuanced insights and major challenges uncovered during Phase 1. Before the scoping workshop, the challenges identified from the four formative work packages (a-d above) and supporting literature were visualised using an online collaborative whiteboard platform (Miro) and then reorganised and refined during two sessions held among the research team with the use of affinity diagramming (29,30). Affinity diagramming is a collaborative sense-making technique that supports a bottom-up approach for qualitative data analysis where data are written on separate sticky notes and categorised/clustered around insights based on their affinity (31–33). At this stage there were 27 challenges which were grouped into three overarching themes: 1) the local healthcare system (10 challenges), 2) the national healthcare system (10 challenges), and 3) maternal and IYC nutrition and feeding practices (9 challenges). Three challenges featured under more than one of the overarching themes, as shown in Supplementary Table 1.

To help define specific issues and narrow down the scope for intervention, we transformed the challenges into opportunities and thematically grouped them, resulting in a list of 19 opportunities (Supplementary Table 1). To assist with prioritising, we asked all research team

For peer review only - http://bmjopen.bmj.com/site/about/guidelines.xhtml

participants to vote independently for their top four (out of 19) most important opportunities from their own perspectives during the scoping workshop. Miro automatically counted the votes and displayed the total votes and the final ranking. Two members who could not attend the workshop voted remotely and these were subsequently added to the total. The votes were used to prioritise the top 10 opportunities (Table 1). We then allocated one of the top-ranking opportunities using the "How Might We" technique (34–36) to each of the four breakout rooms during the scoping workshop. Each breakout room had a mix of interdisciplinary researchers who reflected on their particular challenge/opportunity and identified potential strategies to address them.

After the scoping workshop, we undertook a final round of analysis using affinity diagramming to reduce the number of opportunities that were taken for exploration and scoping with the community participants. Out of the top 10 most voted opportunities from the workshop and the challenges they covered, a final group of four major challenges were selected for consideration for Phase 2:

- High consumption of unhealthy foods, sugar-sweetened beverages, and savoury snacks (fried, salty foods, sweet products) in mothers and IYC.
- Low prevalence of (or difficulties with) iron supplementation in IYC.
- Issues with nutritional counselling and maternal wellbeing.
- No way of tracking progress in relation to the double burden of malnutrition.

Phase 2: Identification of prototypes for nutrition interventions using community-based participatory design

Patient and public involvement

This is a community-based study with the target population being IYC aged 6-23 months. Caregivers of IYC are therefore the public/patient representatives for this study. The participatory design process will include caregivers from the two peri-urban communities in Lima and Huánuco, the healthcare professionals of the participating health centres and the community health agents. Community health agents are members of the community who conduct home visits to mothers with IYC to follow up and promote iron supplementation (including haemoglobin tests or sometimes delivering iron supplements to the home),

For peer review only - http://bmjopen.bmj.com/site/about/guidelines.xhtml

vaccination uptake and attendance at health checks. Throughout this paper, we use 'community stakeholders' to refer to caregivers, healthcare professionals and community health agents.

Aside from caregivers and healthcare professionals directly participating in the research, the study has involved local, regional and national stakeholders since the early stages of developing Phase 1 protocols. Consultation meetings took place with national and regional stakeholders at the inception of the project, and we have continued with regular consultations and presentations of the Phase 1 findings to municipal (regional) government authorities and national government officials in ministries responsible for health and social development. In Huánuco, in addition to the regional authorities, we are also working with the '*Mesa*' (Mesa de Concertación de Lucha contra la Pobreza) which is the coordinating body for civil society organisations.

Through national and local consultations prior to Phase 2, we have been able to develop ways of making the participatory co-design accessible and inclusive for the community stakeholders. For example, workshops will be scheduled at a time of day that is preferred by caregivers to accommodate other commitments (typically 2:30-4:30pm, or 3.00pm-5.00pm). Assistance with transport costs will be provided as that was identified as a barrier to participation. Community health agents will liaise with participants before and after workshops to give reminders of dates, times and location as they are trusted within the communities. Community health agents will also assist with care of IYC and older siblings during workshops to allow caregivers full participation.

In the participatory design phase, community stakeholders will be involved in all steps of the design process from idea generation and storyboarding workshops, to prototype development, formative testing of the interfaces, and piloting.

Study design

Participatory design activities will be led by researchers in HCI and design (NV and DO) with input from the multidisciplinary research team leads from Phase 1 with expertise in nutrition (RPa, HCK, MH, EKR, RPr, PG), nutrition policy (MH, RPr), anthropology and social sciences (RB, HCK, PG, EKR, RPr). The PERUSANO team has previous experience ranging from more than 20 years of research in low- and middle-income community settings

For peer review only - http://bmjopen.bmj.com/site/about/guidelines.xhtml

to mid- and early-career researchers, including doctoral researchers in Peru and in the UK. The team includes researchers fluent in Spanish, English, and both languages (NV, HCK, RPa, DO). The participatory design phase started in June 2022 and will be completed in March 2023.

Methodological framework

Participatory design is a research approach to the development of technology for real-world problems with the main principle to actively involve users in the design decisions of the systems designed, initially rooted at the workplace in Scandinavia (37,38), and later to support everyday life (21,39). Participatory design has shown its potential as a research approach to effectively bridge the gap between computer technologies and health-related interventional research (40), fostering empathy and mutual learning among different stakeholders (41). Participatory design has been used in many different domains including public health, healthcare in clinical and non-clinical settings using a range of different methods to support healthcare technology design (22).

Study settings

The project focuses on the rapidly urbanising population of Peru constituting 78% of the national population (42). The study will take place in peri-urban low-income areas in two regions in Peru (Manchay, Lima and Huánuco city, Huánuco district). In each site, we will work with one principal health centre and one subsidiary health centre (four in total) and the mothers and IYC in the surrounding population.

Study participants and sampling

Co-design workshops of 10-12 participants will include caregivers of IYC, staff of the community health centres with responsibility for nutritional counselling and growth and development checks and the community health agents. Workshops will be held either in the health centre building, or venues close to the health centres. We will engage with community stakeholders to explore, experiment, co-design, co-create and iteratively validate new design ideas to address the four major challenges around IYC nutrition identified in Phase 1.

Sampling

We will invite 10-12 mothers or caregivers to each workshop in each site of Lima and Huánuco (i.e., 20-24 participants in total for each stage of the co-design process) through

For peer review only - http://bmjopen.bmj.com/site/about/guidelines.xhtml

purposive sampling of mothers/caregivers registered at the health centres with IYC. Based on previous experience, we anticipate that not all invited participants will be able to attend on the day and have allowed for up to 50% non-attendance. If there is attrition or drop out between one workshop and the next, new participants from the same community will be invited to take part.

We will purposefully invite two healthcare professionals (including community health agents) to participate from each of the four health centres that are participating in Lima and Huánuco, aiming for at least three healthcare staff to attend each co-design workshop. Health centre participants will be recruited to represent the range of personnel involved in IYC health care namely, nutritionists, nurses responsible for growth and development checks and community health agents. Recruitment of healthcare professionals will be coordinated with the head of the health centres according to their time availability. Caregivers will be recruited by the community health agents who will invite mothers from the list of families assigned to them and send reminders by telephone and WhatsApp. An estimated 95% of households in Peru have access to a mobile phone (43).

Sample size calculation

Community based co-design workshops employ primarily design and qualitative methods. There is no sample size calculation based on a quantitative outcome or measures related to user and design research methods. We aim to recruit a diversity of characteristics in the health professionals (men and women, different job roles, and at different career stages/length of service in the area). Among caregivers, we also aim to recruit participants with a range of characteristics relating age, employment status, education and number of children. We will ensure accountability and rigour through continuous “debate, critique and reflection” (44) between the participants and the research team.

Data collection methods

Prior to the participatory co-design process, the research team will create a set of design artefacts/probes and drawings to actively support the engagement of the participants, such as a visual storyboard with different scenarios (scenario-based challenge cards) reflecting the top challenges from Phase 1, and a collection of ideation/design cards that includes a deck of

For peer review only - http://bmjopen.bmj.com/site/about/guidelines.xhtml

pictorial representations of people, resources and strategies as well as technology coming from the Phase 1 findings, research team suggestions and informed by relevant literature.

We will then conduct a series of four workshops in each site (8 in total), each one of approximately two hours duration on average including a refreshment break. The workshops will take place in parallel in the two study areas led by the same researchers to strengthen methodological consistency and continuity. Different ideas can develop in the two locations, but emerging concepts and design ideas will be cross-referenced across the co-design workshops in each site to allow for complementarity. Workshops will be audio-recorded and visual activities will be recorded by photography and workshop co-created materials. The sequence and purpose of workshops is outlined below:

i. Idea generation workshops

Community stakeholders (e.g. mothers, family members, health facility staff and community health agents) will generate creative and innovative ideas to support healthy diets for IYC including Double Duty Actions (actions to address micronutrient deficiency and anaemia, avoiding excess intake)(8) and will discuss how to implement recommended practices (45). Methods will include using printed sketches with different scenarios and reflecting on the main challenges to healthy dietary practices identified in Phase 1. Scenarios will be read to participants who will then brainstorm using post-it notes and sketch potential solutions (supported by ideation/design cards) or interventions to assist family caregivers, government healthcare centres and staff to enhance nutrition and complementary feeding. Interventions could be digital/non-digital, targeting relevant users (i.e., households, individuals or healthcare professionals) or the wider community or health service environment. At least three design themes will be drawn from the idea generation workshops following collective analysis and evaluation. These will be refined further e.g. using visual sketching techniques for conceptual design (46–48) and to enable future thinking (49,50). A deciding vote will select the preferred design concepts to be considered in the next workshops.

ii. Co-creating future scenarios workshops

Taking a future-oriented perspective (49), community stakeholders will explore and co-create visions of the future/future scenarios through the use of sketching techniques (50) and the 'futures' workshop method (51,52). First, participants will critically reflect on the design ideas/concepts/sketches that emerged from the idea generation workshops. Next, in the 'fantasy' phase, participants will divide into small groups to devise new ideas and visions of

For peer review only - http://bmjopen.bmj.com/site/about/guidelines.xhtml

the future for further development. Use of sketching will promote collaborative reflection, innovation and co-design of the ideal future (53). Finally, participants will discuss the envisaged concepts/scenarios and assign implementation priorities based on suitability to the local communities and select up to three design concepts for further exploration as potential interventions.

iii. Storyboarding workshops

Participants will create storyboards (45) to further develop and visually convey up to three proposed design concepts. They will then elaborate and consolidate the best-case scenario for each concept. After presenting storyboards, participants will discuss the pros and the cons of each concept and a list of key functionalities that stand out for each design concept will be identified.

iv. Early implementation and feedback workshops

Early-stage implementation of the design concepts will take place through design plans, visualisations and creating material artefacts, design probes and intervention prototypes. Prototypes are proposals for future “things-that-are-not-quite-objects-yet” (54), providing insights into what might be possible in the future to current configurations of resources, people and practices. These workshops will gather feedback on the initial design iterations through, e.g., feedback sessions and hands-on activities. At the end, up to one design concept per research site will be selected for further development based on early feedback. High-fidelity prototypes will be implemented and refined after the final workshop and before piloting.

Pilot studies to refine designed intervention prototypes

We will pilot the materials, messages and delivery methods of the intervention prototype in the health facilities or households in the study areas. This may include new approaches to staff training, novel methods for delivering messages for both health service staff or parents/caregivers and/or in the home (e.g., text messaging, games, etc.). Prototypes will be piloted for 1-2 months. Qualitative evaluation will use ethnographic methods, data logging, and pre/post questionnaires and interviews. The evaluations with health services staff will explore acceptability, feasibility and impact potential of the refined version of the generated prototypes. Exit interviews with mothers attending health facilities will be used to evaluate

For peer review only - http://bmjopen.bmj.com/site/about/guidelines.xhtml

their experiences. This project timescale is insufficient to evaluate dietary behaviour changes for IYC feeding, but subsequent funding will be sought to conduct further longer-term evaluations and further redesign and refinement of the prototypes.

Data analysis

Audio-recordings of participatory workshops will be transcribed verbatim in Spanish by the same researcher who facilitates workshops. Observations, written notes and photographs from workshops will be recorded as further supporting materials. Transcripts of workshops will be analysed along with the materials produced, using a collaborative tool (Miro). At each workshop, we will share the summaries and themes with participants that have come from the previous workshop, using drawings and designs to capture ideas and themes, to support discussion, reflection and additional feedback. In depth analysis of data will take place alongside the participatory design process. Coding will be conducted in Spanish primarily by one investigator (DO) who will then check and discuss codes with co-investigators (one expert in HCI (NV) and one nutritionist (RPa)) to reach consensus. Codes will then be translated to English by one of the bilingual researchers. Qualitative data will be analysed using a combination of affinity diagramming to quickly analyse the co-produced materials to identify requirements and complemented by thematic analysis of participants discussions using NVivo software.

Ethics and dissemination

Ethical approval for this study was obtained from the Ethical Review Committee of the Instituto de Investigación Nutricional (IIN) Peru (reference 388-2019/CIEI-IIN), Loughborough University (C19-87) and confirmed by Cardiff University based on local approval in Peru. Written informed consent, and reconsent for repeat workshop participation, will be provided by all participants after receiving written and verbal information about the study. Participants will be informed of the right to withdraw from participation at any stage.

Data (audio-recordings, transcripts, drawings, prototype designs and images) will be stored on a secure workspace at Loughborough University accessible only by investigators and designated research staff during the project. During data collection, information will be stored on encrypted or password protected laptops and tablets in Peru. No personal identifying information will be associated with design outputs, and all drawings, ideas and creative

For peer review only - http://bmjopen.bmj.com/site/about/guidelines.xhtml

output will be stored anonymously. Following the study, data will be stored on the institutional data repository at Loughborough University and made open access after a period of embargo. Data relating to Phase 1 of the project can be accessed at https://doi.org/10.17028/rd.lboro.c.6329105.v1; https://doi.org/10.17028/rd.lboro.c.6329093.v1 and https://doi.org/10.17028/rd.lboro.c.6349121.v1 Publications will follow criteria for reporting qualitative research (COREQ) guidelines (55) and guidelines for reporting patient and public involvement in research (GRIPP2) (56).

Findings of the participatory design process will be disseminated through a deliberative workshop in Lima, Peru. Community stakeholders and policy makers will be invited to the deliberative workshop with study investigators to consider the outcomes of the pilot work. This workshop will share insights from the iterative design process and the developed prototypes. We will reflect on what worked and what did not work during the design activities, identifying factors that facilitated success. Research findings from Phase 1 and 2 will also be disseminated through academic publications. We will seek future opportunities to continue developing the generated prototypes and rolling out the prototypes as a trial intervention with longer-term evaluation to examine the effect on complementary feeding behaviours and dietary outcomes for infant and young child nutrition.

Discussion and conclusions

The first phase of this study identified nutritional challenges relating to stunting, risk of overweight or obesity and anaemia in IYC among low income, peri-urban communities in Peru. From these identified challenges, we prioritised the top four nutritional challenges to take forward to our participatory co-design study (Phase 2). This next phase will involve healthcare professionals, community health agents and caregivers working together to iteratively co-design and test prototypes that aim to address the identified nutritional challenges. The study will offer recommendations for design, research and policy in relation to the co-design of digital health tools in the context of IYC nutrition in Peru. An important consideration in the study is that our formative research was conducted up to March 2020 before the COVID-19 pandemic, and our participatory-co-design phase started in June 2022 when most restrictions relating to COVID-19 were lifted. The intervening period may have influenced caregiver and healthcare professionals' ideas and perceived needs for healthy nutrition of IYC. Working across two languages, English and Spanish, also adds some

For peer review only - http://bmjopen.bmj.com/site/about/guidelines.xhtml

complexities to the co-design process. We also recognise that the participatory co-design work conducted in Lima and Huánuco may not capture the nutritional challenges for all urban contexts in Peru, and future work would need to examine how our co-design approach and emerging prototypes could be adapted for other urban and rural environments.

Authors' contributions

EKR, HCK, NV, RPa, RB, MH, PG designed and led the development of the funded grant proposal, EKR, RPa, HCK, NV, RB, MH, PG, RPr, and DO developed detailed protocols for Phase 1 and the participatory co-design (Phase 2). All authors contributed to writing the manuscript and had critical input on the final version.

Contribution to public involvement

We thank the healthcare professionals from the participating health centres and mothers with infants and young children for their active participation in the development of this protocol.

Funding statement

This study was supported by the UK Medical Research Council (MR/S024921/1) and CONCYTEC/ PROCIENCIA Perú (032-2019-FONDECYT) through the Newton Fund.

Competing interests' statement

The authors declare no competing interests.

Word count: 3993

Acknowledgements We gratefully acknowledge the contribution of all the investigators and field and research assistants on the PERUSANO project.

For peer review only - http://bmjopen.bmj.com/site/about/guidelines.xhtml

References

1. World Health Organization. Complementary Feeding of Young Children in Developing Countries: A Review of Current Scientific Knowledge. Geneva; 1998.

2. World Health Organization. Guiding Principles for Feeding Non-Breastfed Children 6-24 Months of Age. Geneva; 2005.

3. WHO, UNICEF, USAID, FANTA 2, UC Davis, AED. Indicators for Assessing Infant and Young Child Feeding Practices, Part 1: Definitions. Geneva; 2008.

4. Hawkes C, Harris J, Gillespie S. Changing diets: Urbanization and the nutrition transition. 2017 Glob Food Policy Rep. 2017;34–41.

5. Pries AM, Huffman SL, Champeny M, Adhikary I, Benjamin M, Coly AN, et al. Consumption of commercially produced snack foods and sugar-sweetened beverages during the complementary feeding period in four African and Asian urban contexts. Matern Child Nutr. 2017 Oct;13:e12412.

6. Rousham EK, Goudet S, Markey O, Griffiths P, Boxer B, Carroll C, et al. Unhealthy Food and Beverage Consumption in Children and Risk of Overweight and Obesity: A Systematic Review and Meta-Analysis. Adv Nutr. 2022 Oct 2;13(5):1669–96.

7. Lutter CK, Grummer-Strawn L, Rogers L. Complementary feeding of infants and young children 6 to 23 months of age. Nutr Rev. 2021 Jul 7;79(8):825–46.

8. WHO. Double-duty actions: policy brief. Geneva; 2017.

9. Development Initiatives. 2020 Global Nutrition Report: Action on equity to end malnutrition. Bristol, UK; 2020.

10. INEI. Encuesta Demográfica y de Salud Familiar (ENDES) 2020. Lima; 2021.

11. Huicho L, Huayanay-Espinoza CA, Herrera-Perez E, Segura ER, Niño de Guzman J, Rivera-Ch M, et al. Factors behind the success story of under-five stunting in Peru: a district ecological multilevel analysis. BMC Pediatr. 2017 Dec 19;17(1):29.

12. Ministry of Health G of P. Plan nacional para la reducción y control de la anemia materno infantil y la desnutrición crónica infantil en el Perú: 2017 – 2021. 2017.

For peer review only - http://bmjopen.bmj.com/site/about/guidelines.xhtml

13. INEI. Encuesta Demográfica y de Salud Familiar (ENDES) 2017. Lima; 2017.

14. Penny ME, Michelle Jimenez M, Margot Marin R. Early rapid weight gain and subsequent overweight and obesity in middle childhood in Peru. BMC Obes. 2016 Dec 13;3(1):55.

15. Creed-Kanashiro H, Wasser HM, Bartolini R, Goya C, Bentley ME. Formative research to explore the acceptability and use of infant food grinders for the promotion of animal source foods and micronutrient powders in rural Peru. Matern Child Nutr. 2018 Oct 2;14(4):e12600.

16. Holeman I, Kane D. Human-centered design for global health equity. Inf Technol Dev. 2020 Jul 2;26(3):477–505.

17. Papoutsi C, Wherton J, Shaw S, Morrison C, Greenhalgh T. Putting the social back into sociotechnical: Case studies of co-design in digital health. J Am Med Informatics Assoc. 2021 Feb 15;28(2):284–93.

18. Unertl KM, Schaefbauer CL, Campbell TR, Senteio C, Siek KA, Bakken S, et al. Integrating community-based participatory research and informatics approaches to improve the engagement and health of underserved populations. J Am Med Informatics Assoc. 2016 Jan 1;23(1):60–73.

19. Hussain S, Sanders EB-N, Steinert M. Participatory design with marginalized people in developing countries: Challenges and opportunities experienced in a field study in Cambodia. Int J Des. 2012;6(2):91–109.

20. Sanders EBN, Stappers PJ. Probes, toolkits and prototypes: Three approaches to making in codesigning. CoDesign. 2014 Jan 2;10(1):5–14.

21. Halskov K, Hansen NB. The diversity of participatory design research practice at PDC 2002-2012. Int J Hum Comput Stud. 2015 Feb;74:81–92.

22. Kanstrup AM, Madsen J, Nøhr C, Bygholm A, Bertelsen P. Developments in participatory design of health information technology - A review of PDC publications from 1990 - 2016. Particip Des Heal Inf Technol. 2017;233:1–13.

23. Andía D, Charca S, Reynolds-Cuéllar P, Noel J. Community-oriented engineering co-design: case studies from the Peruvian Highlands. Humanit Soc Sci Commun. 2022 Sep 14;9(1):311.

For peer review only - http://bmjopen.bmj.com/site/about/guidelines.xhtml

24. Lasage R, Muis S, Sardella CSE, van Drunen MA, Verburg PH, Aerts JCJH. A Stepwise, participatory approach to design and implement community based adaptation to drought in the Peruvian Andes. Sustain. 2015 Feb 10;7(2):1742–73.

25. Smits KM, McDonald L, Smith NM, Gonzalez F, Lucena J, Martinez G, et al. Voces Mineras: Clarifying the future of artisanal and small-scale mining collaborations. Extr Ind Soc. 2020 Jan;7(1):68–72.

26. Lazo-Porras M, Perez-Leon S, Cardenas MK, Pesantes MA, Miranda JJ, Suggs LS, et al. Lessons learned about co-creation: developing a complex intervention in rural Peru. Glob Health Action. 2020 Dec 31;13(1):1754016.

27. Greene MC, Bonz A, Cristobal M, Vega C, Andersen LS, Angulo A, et al. Evaluating the feasibility of a group psychosocial intervention for migrant and host community women in Ecuador and Panamá: protocol for a multi-site feasibility cluster trial. Pilot Feasibility Stud. 2022 Dec 15;8(1):126.

28. Braa J, Titlestad OH, Sæbø J. Participatory health information systems development in Cuba. In: Proceedings of the eighth conference on Participatory design Artful integration: interweaving media, materials and practices - PDC 04. New York, New York, USA: ACM Press; 2004. p. 53.

29. Hanington BM, Martin B. Universal Methods of Design: 125 Ways to Research Complex Problems, Develop Innovative Ideas, and Design Effective Solutions. Rockport International; 2019.

30. Harrington HJ. Affinity Diagrams. In: The Innovation Tools Handbook, Volume 2. Productivity Press; 2016. p. 45–54.

31. Remy C, Harboe G, Frich J, Biskjaer MM, Dalsgaard P. Challenges and Opportunities in the Design of Digital Distributed Affinity Diagramming Tools. In: European Conference on Cognitive Ergonomics 2021. New York, NY, USA: ACM; 2021. p. 1–5.

32. Holtzblatt K, Beyer H. Contextual design: defining customer-centered systems. Elsevier; 1997.

33. Harboe G, Huang EM. Real-World Affinity Diagramming Practices. In: Proceedings of the 33rd Annual ACM Conference on Human Factors in Computing Systems. New

For peer review only - http://bmjopen.bmj.com/site/about/guidelines.xhtml

York, NY, USA: ACM; 2015. p. 95–104.

34. Carroll M, Goldman S, Britos L, Koh J, Royalty A, Hornstein M. Destination, Imagination and the Fires Within: Design Thinking in a Middle School Classroom. Int J Art Des Educ. 2010 Mar 15;29(1):37–53.

35. Chatty T, Qu Y, Ba-Sabaa HH, Murnane EL. Examining the user experience of life cycle assessment tools and their ability to cater to ecodesign in early-stage product development practice. Proc Des Soc. 2021 Aug 27;1:1441–50.

36. Kim S, Jung DHS, Upender A, Claire S, Esfandiari I. Human-Food Interaction Framework: A New Design Tool Used to Understand Amateur Home Cooks' Needs. In: International Conference on Human-Computer Interaction. Springer; 2019. p. 9–14.

37. Bødker S, Ehn P, Knudsen J, Kyng M, Madsen K. Computer support for cooperative design (invited paper). In: Proceedings of the 1988 ACM conference on Computer-supported cooperative work - CSCW '88. New York, New York, USA: ACM Press; 1988. p. 377–94.

38. Bφdker S, Grφnbæk K, Kyng M. Cooperative design: techniques and experiences from the Scandinavian scene. In: Readings in Human–Computer Interaction. Morgan Kaufmann; 1995. p. 215–24.

39. Bødker S. When second wave HCI meets third wave challenges. In: ACM International Conference Proceeding Series. New York, New York, USA: ACM Press; 2006. p. 1–8.

40. Clemensen J, Larsen SB, Kyng M, Kirkevold M. Participatory design in health sciences: Using cooperative experimental methods in developing health services and computer technology. Qual Health Res. 2007 Jan 1;17(1):122–30.

41. Akoglu C, Dankl K. Co-creation for empathy and mutual learning: a framework for design in health and social care. CoDesign. 2021 Jul 3;17(3):296–312.

42. INEI. Encuesta Demográfica y de Salud Familiar (ENDES) 2019. Lima; 2020.

43. INEI. Evolución de la Pobreza Monetaria 2009–2020: Encuesta Nacional de Hogares, 2010–2020 (Informe Técnico). Lima; 2020.

44. Frauenberger C, Good J, Fitzpatrick G, Iversen OS. In pursuit of rigour and

For peer review only

For peer review only - http://bmjopen.bmj.com/site/about/guidelines.xhtml

accountability in participatory design. Int J Hum Comput Stud. 2015 Feb;74:93–106.

45. Erickson T. Design as Storytelling. In: Interactions. ACM Publications; 1996. p. 30–5.

46. McGown A, Green G, Rodgers PA. Visible ideas: Information patterns of conceptual sketch activity. Des Stud. 1998;19(4):431–53.

47. Rodgers PA, Green G, McGown A. Using concept sketches to track design progress. Des Stud. 2000;21(5):451–64.

48. Chen S. The Design Studio Approach. In: The Design Imperative. Champaign, Illinois: Palgrave Macmillan; 2019. p. 55–70.

49. Kjærsgaard MG, Halse J, Smith RC, Vangkilde KT, Binder T, Otto T. Introduction: design anthropological futures. In: Design Anthropological Futures. Routledge; 2020. p. 1–16.

50. Sturdee M, Lindley J. Sketching & drawing as future inquiry in HCI. ACM Int Conf Proceeding Ser. 2019;1–10.

51. Kensing F, Madsen H. Generating Visions: Future Workshops and Metaphorical. In: Design at Work: Cooperative Design of Comuter Systems. CRC Press; 1991.

52. Jungk K, Müllert N. No Title. In: Future Workshops: How to create desirable futures. Institute for Social Inventions; 1987.

53. Sanders EBN, Stappers PJ. Co-creation and the new landscapes of design. CoDesign. 2008 Mar;4(1):5–18.

54. Corsín Jiménez A. Introduction: The prototype: more than many and less than one. J Cult Econ. 2014 Oct 2;7(4):381–98.

55. Tong A, Sainsbury P, Craig J. Consolidated criteria for reporting qualitative research (COREQ): A 32-item checklist for interviews and focus groups. Int J Qual Heal Care. 2007 Sep 16;19(6):349–57.

56. Staniszewska S, Brett J, Simera I, Seers K, Mockford C, Goodlad S, et al. GRIPP2 reporting checklists: Tools to improve reporting of patient and public involvement in research. BMJ. 2017 Aug 2;358:j3453.

For peer review only - http://bmjopen.bmj.com/site/about/guidelines.xhtml

Table 1. Top ten opportunities for intervention in infant and young child (IYC) nutrition identified and prioritised through formative research and scoping workshops to take forward to the participatory design phase

**Opportunity statements identified and prioritised by the research team†**
Reduce the consumption of unhealthy foods and beverages amongst mothers and IYC (6-23 months) to improve the overall quality of diet/feeding practices
Develop strategies to reduce inequalities to protect vulnerable populations in relation to the double burden of malnutrition
Improve emotional and psychological wellbeing for mothers to reduce its impact on IYC nutrition
Increase/enhance maternal nutrition knowledge on appropriate feeding practices (e.g., iron supplementation, complementary feeding, breastfeeding)
Help to implement governmental policies for early childhood education services to provide and promote healthy food choices
Increase adherence to iron supplementation for IYC
Enhance the uptake, access, and intercultural approaches to improve maternal and child care services
Enhance counselling skills for healthcare professionals
Improve nutrition counselling during pregnancy and from birth onwards
Address the challenges in coordinating the implementation and evaluation of policy across different levels of governmental institutions in relation to the double burden of malnutrition

†The full list of opportunities which were voted on to prioritise the 10 ten opportunities are listed in Supplementary Table 1.

For peer review only - http://bmjopen.bmj.com/site/about/guidelines.xhtml

Figure legends

Figure 1: Overview of the PERUSANO study; Phase 1 summarises the formative research objectives and Phase 2 outlines the protocol for the participatory co-design

Figure footnote: FFQ= food frequency questionnaire; IYC= infants and young children

For peer review only

For peer review only - http://bmjopen.bmj.com/site/about/guidelines.xhtml

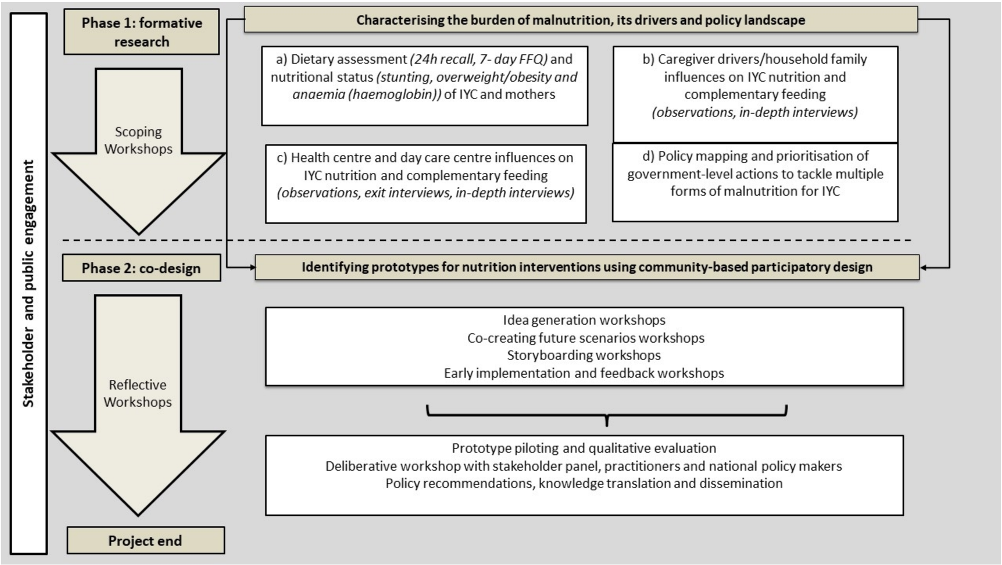

Figure 1: Overview of the PERUSANO study; Phase 1 summarises the formative research objectives and Phase 2 outlines the protocol for the participatory co-design

Figure footnote: FFQ= food frequency questionnaire; IYC= infants and young children

338x190mm (96 x 96 DPI)

Designing intervention prototypes to improve infant and young child nutrition in Peru: A participatory design study protocol

E. K. Rousham, R. Pareja, H.M. Creed-Kanashiro, R. Bartolini, R. Pradeilles, D. Ortega-Roman, M. Holdsworth, P. Griffiths, N. Verdezoto

Supplementary Table 1: Summary of the 27 identified nutrition challenges, and 19 opportunities identified for infants and young children (IYC) aged 6-23 months in two peri-urban communities in Peru prior to the participatory co-design phase of the study

Overarching theme	Challenges identified in the formative research‡	Opportunity statements derived from the challenges
Improve the healthiness of maternal diets and infant & young child feeding practices	1. High consumption of sugar-sweetened beverages in mothers and IYC	1. Reduce the consumption of unhealthy foods and beverages amongst mothers and IYC to improve the overall quality of diet and feeding practices
	2. High consumption of unhealthy foods (savoury snacks/fried foods, sweet products) in mothers and IYC	
	3. Sub-optimal dietary diversity, particularly around animal-source foods and fruit and vegetable consumption, due to small quantities consumed	2. Improve the diversity of diets for mothers and IYC
	4. Inadequate engagement with responsive feeding in the home	3. Increase the engagement with responsive feeding practices at home
	5. Low prevalence of exclusive breastfeeding under six months, including early introduction of complementary foods†	4. Increase the prevalence of exclusive breastfeeding for infants under 6 months
	6. Low compliance with taking iron supplements in IYC†	5. Increase adherence to iron supplementation
	7. Issues with emotional and psychological wellbeing of mothers which impacts on the caring and feeding practices for the child	6. Improve emotional and psychological wellbeing for mothers to reduce its impact on IYC nutrition
	8. Mothers' understanding of nutrition knowledge and perception and practices around infant nutrition	7. Increase/enhance maternal nutrition knowledge on appropriate feeding practices (e.g., iron supplementation, complementary feeding, breastfeeding)
	6. Low compliance with taking iron supplements in IYC†	
	5. Low prevalence of exclusive breastfeeding under 6 months (which means that other liquids and complementary foods are introduced early)†	
	9. Challenges with regards to maternal time use in the home and outside the home	8. Improve the maternal care experience and practices at home/in everyday life -- e.g., tensions with work and time allocation
Enhance the local systems in the community to improve maternal and child nutrition	10. Lack of physical resources and materials	9. Enhance the physical and/or material resources within healthcare settings
	11. Issues with the organisation of consultations, including health centre structures and workflow	10. Optimise the organisation of consultations and workflow within healthcare settings
	12. Health service recommendations do not take into account the socio-cultural practices and norms of caregivers	11. Enhance the uptake, access, and intercultural approaches to improve maternal and child care services

For peer review only - http://bmjopen.bmj.com/site/about/guidelines.xhtml

	13. Issues with the uptake and access to health services for IYC†	
	14. Issues with regards to counselling approaches and staff skills within health centres	12. Enhance counselling skills for healthcare professionals
	15. Community-based activities for breastfeeding outreach and support have national coverage	13. Facilitate community-based activities to enhance breastfeeding practices
	16. Inadequate counselling for complementary feeding	14. Improve nutrition counselling during pregnancy and from birth onwards
	17. Inadequate nutritional counselling for pregnant women	
	18. Restriction on infant formula promotion in ante-natal and post-natal settings	15. Promote the reduction of the use and promotion infant formula in antenatal and postnatal care settings
	19. Government policy should ensure food services in early childhood education provide and promote healthy food choices (e.g. via canteens, food at events, fundraising, promotions, vending machines)†	16. Help to implement governmental policies for early childhood education services to provide and promote healthy food choices
Support the national system to improve maternal and child nutrition policy implementation	20. Funding for interventions and policies to reduce obesity, stunting and iron deficiency anaemia in IYC	17. Seek funding opportunities to develop interventions and policies to address the double burden of malnutrition
	21. Monitoring systems particularly in vulnerable young children's populations for the five double duty actions and to track progress towards reducing nutritional inequalities	18. Develop strategies to reduce inequalities to protect vulnerable populations in relation to the double burden of malnutrition
	22. Reduce inequalities or protect vulnerable populations in relation to obesity, stunting and iron deficiency anaemia in IYC	
	23. Lack of use of ICT interventions for the double burden of malnutrition in low and middle income country settings*	
	13. Issues with the uptake and access to health services for IYC†	
	24. Restrict commercial influences on the development of policies related to preventing obesity, stunting and iron deficiency anaemia in IYC	
	25. Robust coordination to ensure alignment, and integration of policies in obesity, stunting and iron deficiency anaemia in IYC across departments and levels of government (national and local)	19. Address the challenges in coordinating the implementation and evaluation of policy across different levels of governmental institutions in relation to the double burden of malnutrition
	26. Lack of coherence in the policy environment (e.g. lack of a holistic nutrition approach, no attention to the double burden of malnutrition	
	19. Government policy should ensure food services in early childhood education provide and promote healthy food choices (e.g. via canteens, food at events, fundraising, promotions, vending machines)†	

iew only

	27. Low adherence to taking iron supplements, reasons include fear of side effects and difficulties of administering at home, and low quality iron supplements that government offers	

‡Shading represents the source of each challenge from Phase 1 work packages. Key: no fill = work package a) Dietary assessment and nutritional status of IYC and mothers; light grey = work package b) Caregiver drivers and household or family influences on IYC nutrition and complementary feeding; mid-grey = work package c) Health centre and day care centre influences on IYC nutrition and complementary feeding; dark-grey = work package d) Policy mapping and prioritisation of government-level actions to tackle multiple forms of malnutrition for IYC.

†Challenges that appeared under more than one overarching theme

*Challenge identified from literature review

For peer review only

BMJ Open

Designing intervention prototypes to improve infant and young child nutrition in Peru: A participatory design study protocol

Journal:	*BMJ Open*
Manuscript ID	bmjopen-2022-071280.R1
Article Type:	Protocol
Date Submitted by the Author:	02-Nov-2023

Complete List of Authors:	Rousham, Emily; Loughborough University<br>Pareja, Rossina G. ; Instituto de Investigación Nutricional<br>Creed-Kanashiro, H.M.; Instituto de Investigación Nutricional<br>Bartolini, R.; Instituto de Investigación Nutricional<br>Pradeilles, Rebecca; Loughborough University<br>Ortega-Roman, Deysi; Cardiff University<br>Holdsworth, Michelle; IRD, NUTRIPASS<br>Griffiths, Paula; Loughborough University<br>Verdezoto, Nervo; Cardiff University

**Primary Subject Heading**:	Global health
Secondary Subject Heading:	Public health, Qualitative research
Keywords:	NUTRITION & DIETETICS, Community child health < PAEDIATRICS, PUBLIC HEALTH, Health policy < HEALTH SERVICES ADMINISTRATION & MANAGEMENT

SCHOLARONE™
Manuscripts

Designing intervention prototypes to improve infant and young child nutrition in Peru: A participatory design study protocol

E. K. Rousham[1*], R. Pareja[2*], H.M. Creed-Kanashiro[2], R. Bartolini[2], R. Pradeilles[1], D. Ortega-Roman[3], M. Holdsworth[4], P. Griffiths[1], N. Verdezoto[3]

*Authors contributed equally

Authors' affiliations

[1] Centre for Global Health and Human Development, School of Sport, Exercise and Health Sciences, Loughborough University, Loughborough, UK

[2] Instituto de Investigación Nutricional, Lima, Perú

[3] School of Computer Science and Informatics, Cardiff University, Cardiff, UK

[4] UMR MoISA (Montpellier Interdisciplinary Centre on Sustainable Agri-Food Systems), (Univ Montpellier, CIRAD, CIHEAM-IAMM, INRAE, Institut Agro, IRD), Montpellier, France

Corresponding authors

Prof E. K. Rousham, Centre for Global Health and Human Development, School of Sport, Exercise and Health Sciences, Loughborough University, Loughborough, LE11 3TU, UK. e.k.rousham@lboro.ac.uk

And

Dr N. Verdezoto, School of Computer Science and Informatics, Cardiff University, Cardiff, CF24 4AG, UK. verdezotodiasn@cardiff.ac.uk

For peer review only

Abstract

Introduction: Nutrition during the complementary feeding period (6-23 months) is critical to ensure optimal growth and reduce the risk of diet-related disease across the life course. Strategies to reduce multiple forms of malnutrition (stunting, overweight/obesity and anaemia) in infants and young children (IYC) are a key priority in low- and middle-income countries, including Peru. This study aims to co-design and develop prototypes for interventions to address the multiple forms of malnutrition in IYC in urban Peru, using a participatory design approach.

Methods and analysis: The study will be based within peri-urban communities in two areas of Peru (Lima and Huánuco city). Following the identification of key nutritional challenges for IYC aged 6-23 months through formative research (Phase 1), we will conduct a series of workshops bringing together healthcare professionals from government health centres and caregivers of IYC aged 6-23 months. Workshops (on idea generation; creating future scenarios; storyboarding, and early implementation and feedback) will take place in parallel in the two study areas. Through these workshops, we will engage with community participants to explore, experiment, co-design, and iteratively validate new design ideas to address the challenges around IYC complementary feeding from Phase 1. Workshop outputs and transcripts will be analysed qualitatively using affinity diagramming and thematic analyses. The intervention prototypes will be evaluated qualitatively and piloted with the participating communities.

Ethics and dissemination: Ethical approval for this study was obtained from the Ethical Review Committee of the Instituto de Investigación Nutricional (IIN) Peru (388-2019/CIEI-IIN), Loughborough University (C19-87) and confirmed by Cardiff University. Findings of the participatory design process will be disseminated through a deliberative workshop in Lima, Peru with national and regional government stakeholders, as well as participants and researchers involved in the design process. Further dissemination will take place through policy briefs, conferences and academic publications.

For peer review only

Strengths and limitations of this study

- Participatory co-design is an important way of incorporating the voice of the community in the design and development of health interventions.
- This participatory study will bring together healthcare providers and health service users in collaborative co-design workshops to promote active community involvement in infant and young child nutrition, and mutual learning for all participants including the researchers.
- The co-designed prototypes will be tailored to the nutritional challenges for infants and young children in peri-urban Peru.
- The experiences and ideas expressed by participants may be influenced by the impact of the COVID-19 pandemic on health services and access to healthcare in the previous two years.

For peer review only

For peer review only - http://bmjopen.bmj.com/site/about/guidelines.xhtml

Introduction

Nutrition during the complementary feeding period (6-23 months) is critical to ensure optimal growth and reduce the risk of diet-related diseases across the life course. In low-resource settings, the prevention of undernutrition in the form of stunting, wasting or micronutrient deficiencies has been the traditional focus of complementary feeding guidance (1–3). However, as populations go through dietary and nutrition transitions, infant and young child (IYC) diets are changing with increased consumption of sugar, fats and oils, refined grains and highly processed foods, bringing the additional risk of excess energy intake leading to overweight and obesity (4–7). This in turn leads to increased risk of diet-related diseases in later life. Strategies are now needed to address coexisting forms of malnutrition in infancy and childhood including stunting, micronutrient deficiencies and overweight/obesity (7–9).

Peru faces the public health challenge of multiple burdens of malnutrition with a high prevalence of iron-deficiency anaemia, stunting (low height-for-age) and a rapidly increasing prevalence of overweight and obesity (10). Peru has successfully reduced the rate of stunting in children under 5 years from 23.2% in 2010 to 12.1% in 2020 (10). This success has been attributed to anti-poverty policies, multi-sectoral government working and implementation of cross-cutting interventions in the poorest areas (11). In contrast, anaemia prevalence has changed little among children aged 6–36 months from 43.5% in 2015 to 40.0% in 2020 (INEI, 2015, 2021), despite this being a national priority. Since 2017, government strategies to address anaemia have focussed on national distribution of multi-micronutrient powders and iron supplementation for IYC through health services and promotion of iron-rich foods (12).

Additional concerns focus on the increasing prevalence of overweight and obesity in Peru, affecting 37.4% of children age 5-9 years (13). Cohort studies in Peru have shown rapid weight gain in infancy is associated with overweight and obesity at 8 years (14). Feeding practices in infancy or early childhood also shape future dietary behaviours, such that unhealthy complementary feeding practices may predispose to unhealthy diets in later life (6).

Although previous research has examined the influence of socio-cultural contexts on complementary feeding practices in relation to undernutrition (15), less attention has been given to exploring these influences on excess energy intake and overweight during early life in Peru. Similarly, there are no integrated policies or interventions to prevent overweight alongside iron and micronutrient deficiencies in IYC.

For peer review only - http://bmjopen.bmj.com/site/about/guidelines.xhtml

Socio-technical approaches to healthcare technology design and implementation, such as human-centered design, co-design and community-based participatory research, have led to an increasing involvement of multiple stakeholders in the design process (16–18). Such methods can help multiple stakeholders, including community members, to express their thoughts, share ideas, experiment and actively participate (19) throughout the design process (20). However, most of these socio-technical initiatives have taken place in the Global North (21,22) highlighting the need for the development of new methods and tools to engage with Global South communities. While some previous studies have applied participatory co-design methods in Latin American or Peruvian contexts (23–27), few have involved technology in healthcare settings (28).

The aim of this study is to co-design and develop prototypes for interventions to address the multiple forms of malnutrition coexisting in IYC in Peru, using a participatory design approach. This co-design follows on from formative research in the communities and health services that used qualitative and quantitative data as well as policy mapping to assess the extent of malnutrition in peri-urban communities, underlying contributors to malnutrition, and the key nutritional challenges identified by researchers and community participants.

Methods and analysis

Study background and Phase 1

The PERUSANO study is an interdisciplinary project which aims to address multiple forms of malnutrition among IYC aged 6-23 months in peri-urban communities in Peru. This protocol follows on from the first phase of research in the PERUSANO project. The formative research (Phase 1) aimed to identify the prevalence of all forms of malnutrition as well as the challenges and facilitators of healthy IYC feeding practices. This informed the second phase of the project that will take a co-design approach with community participants (healthcare professionals, mothers and family caregivers, and other community actors) to scope opportunities to address the identified IYC feeding challenges. The wider PERUSANO project team includes a cross-cultural and multidisciplinary group of researchers (seven from Peru and ten from UK/France) with backgrounds in public health, nutrition, social sciences, and human-computer interaction (HCI). Phase 1 objectives were achieved through four work packages: (a) cross-sectional, quantitative assessment of dietary intake (via a 7 day food-frequency questionnaire (FFQ) for mothers and a quantitative 24 hour dietary recall for IYC) and nutritional status (weight, length/height and haemoglobin concentration) of IYC and

For peer review only - http://bmjopen.bmj.com/site/about/guidelines.xhtml

mothers alongside a household socio-demographic survey (n=244); (b) household in-depth qualitative studies (semi-structured interviews, observations) of caregiver drivers of IYC feeding (e.g., feeding style, food attributes) (c) interviews and observations of healthcare professionals responsible for nutritional counselling and well-baby clinics and, (d) policy mapping and prioritisation of government-level actions that exist to tackle multiple forms of malnutrition for IYC with sixteen national experts. An overview of the study design with Phase 1 work packages and Phase 2 components is shown in **Figure 1**.

Phase 1 started in December 2019 and was interrupted by the COVID-19 pandemic in March 2020. COVID-19 restrictions in Peru prevented face-to-face workshops or field work taking place from March 2020 to May 2022. During this time, we analysed data from Phase 1 and continued meetings with researchers and stakeholders via online platforms. An 11 month no-cost extension to the project was granted by funders due to the impact of COVID-19 which enabled face-to-face data collection to commence again in June 2022.

End of Phase 1 (Pre-Design): Identification of key infant and young child nutritional challenges for participatory co-design

In July 2021, we conducted an online scoping workshop with 19 members of the interdisciplinary team, where researchers presented the nuanced insights and major challenges uncovered during Phase 1. Before the scoping workshop, the challenges identified from the four formative work packages (a-d above) and supporting literature were visualised using an online collaborative whiteboard platform (Miro) and then reorganised and refined during two sessions held among the research team with the use of affinity diagramming (29,30). Affinity diagramming is a collaborative sense-making technique that supports a bottom-up approach for qualitative data analysis where data are written on separate sticky notes and categorised/clustered around insights based on their affinity (31–33). At this stage there were 27 challenges which were grouped into three overarching themes: 1) the local healthcare system (10 challenges), 2) the national healthcare system (10 challenges), and 3) maternal and IYC nutrition and feeding practices (9 challenges). Three challenges featured under more than one of the overarching themes, as shown in Supplementary Table 1.

To help define specific issues and narrow down the scope for intervention, we transformed the challenges into opportunities and thematically grouped them, resulting in 19 opportunities (Supplementary Table 1). To assist with prioritising, we asked all research team participants to vote independently for their top four (out of 19) most important opportunities from their

For peer review only - http://bmjopen.bmj.com/site/about/guidelines.xhtml

own perspectives during the scoping workshop. Miro automatically counted the votes and displayed the total votes and the final ranking. Two members who could not attend the workshop voted remotely and these were subsequently added to the total. The votes were used to select the top 10 opportunities (Table 1). We then allocated one of the top-ranking opportunities using the "How Might We" technique (34–36) to each of the four breakout rooms during the scoping workshop. Each breakout room had a mix of interdisciplinary researchers who then reflected on one of the top particular challenge/opportunity and identified potential strategies to address them.

After the scoping workshop, we undertook a final round of analysis using affinity diagramming to select opportunities to take forward for exploration and scoping with the community participants. Out of the top 10 most voted opportunities and their associated challenges, a final group of four major challenges were selected for Phase 2:

- High consumption of unhealthy foods, sugar-sweetened beverages, and savoury snacks (fried, salty foods, sweet products) in mothers and IYC.
- Low prevalence of (or difficulties with) iron supplementation in IYC.
- Issues with nutritional counselling and maternal wellbeing.
- No way of tracking the double burden of malnutrition.

Phase 2: Identification of prototypes for nutrition interventions using community-based participatory design

Patient and public involvement

The study has involved local, regional and national stakeholders since the early stages of developing Phase 1 protocols. Consultation meetings took place with national and regional stakeholders at the inception of the project, and we have continued with regular consultations and presentations of the Phase 1 findings to municipal (regional) government authorities and national government officials in ministries responsible for health and social development. In Huánuco, in addition to the regional authorities, we are also working with the '*Mesa*' (Mesa de Concertación de Lucha contra la Pobreza) which is the coordinating body for civic society organisations.

For peer review only - http://bmjopen.bmj.com/site/about/guidelines.xhtml

Through national and local consultations prior to Phase 2, we have been able to develop ways of making the participatory co-design accessible and inclusive. For example, workshops will be scheduled at a time of day that is preferred by caregivers to accommodate other commitments (typically 2:30-4:30pm, or 3.00pm-5.00pm). Assistance with transport costs will be provided as that was identified as a barrier to participation. Community health agents will liaise with participants before and after workshops to give reminders of dates, times and location as they are trusted within the communities. Community health agents will also assist with care of IYC and older siblings during workshops to allow caregivers full participation.

Study design

Participatory design activities will be led by researchers in HCI and design (NV and DO) with input from the multidisciplinary research team leads from Phase 1 with expertise in nutrition (RPa, HCK, MH, EKR, RPr, PG), nutrition policy (MH, RPr), anthropology and social sciences (RB, HCK, PG, EKR, RPr). The PERUSANO team has previous experience ranging from more than 20 years of research in low- and middle-income community settings to mid- and early-career researchers, including doctoral researchers in Peru and in the UK. The team includes researchers fluent in Spanish, English, and both languages (NV, HCK, RPa, DO). The participatory design phase started in June 2022 and will be completed in March 2023.

Methodological framework

Participatory design is a research approach to the development of technology for real-world problems with the main principle to actively involve users in the design decisions of the systems designed, initially rooted within the workplace in Scandinavia (37,38), and later to support everyday life (21,39). Participatory design has shown its potential as a research approach to effectively bridge the gap between computer technologies and health-related interventional research (40), fostering empathy and mutual learning among different stakeholders (41). Participatory design has been used in many different domains including public health and healthcare in clinical and non-clinical settings using a range of different methods to support healthcare technology design (22).

Study settings

For peer review only - http://bmjopen.bmj.com/site/about/guidelines.xhtml
For peer review only

The project focuses on the rapidly urbanising population of Peru constituting 78% of the national population (42). The study will take place in peri-urban, low-income areas in two regions in Peru (Manchay, Lima and Huánuco city, Huánuco district). In each site, we will work with one principal health centre and one subsidiary health centre (four in total) and the mothers and IYC in the surrounding population.

Study participants

The participatory design process will include caregivers from the two peri-urban communities in Lima and Huánuco, the healthcare professionals of the participating health centres and the community health agents. Community health agents are members of the community who conduct home visits to mothers with IYC to follow up and promote iron supplementation (including haemoglobin tests or sometimes delivering iron supplements to the home), vaccination uptake and attendance at health checks. Throughout this paper, we use 'community participants' to refer to caregivers, healthcare professionals and community health agents. Co-design workshops will include caregivers of IYC, staff of the community health centres with responsibility for nutritional counselling and growth and development checks and the community health agents. Workshops will be held either in the health centre building, or venues close to the health centres. We will engage with community participants to explore, experiment, co-design, co-create and iteratively validate new design ideas to address the four major challenges around IYC nutrition identified in Phase 1. The community-based co-design workshops will take place in parallel recognising that the socioeconomic and cultural context as well as digital literacy in each study location might differ and influence differently the outcomes of the co-design process (43,44).

For peer review only

Sampling

We will invite 10-12 mothers or caregivers to each workshop in each site of Lima and Huánuco (i.e., 20-24 participants in total for each stage of the co-design process) through purposive sampling of mothers/caregivers registered at the health centres with IYC. Based on previous experience, we anticipate that not all invited participants will be able to attend on the day and have allowed for up to 50% non-attendance. If there is attrition or drop out between one workshop and the next, new participants from the same community will be invited to take part.

For peer review only - http://bmjopen.bmj.com/site/about/guidelines.xhtml

We will purposefully invite two healthcare professionals (including community health agents) to participate from each of the four health centres that are participating in Lima and Huánuco, aiming for at least three healthcare staff to attend each co-design workshop. Health centre participants will be recruited to represent the range of personnel involved in IYC health care namely, nutritionists, nurses responsible for growth and development checks and community health agents. Recruitment of healthcare professionals will be coordinated with the head of the health centres according to their time availability. Caregivers will be recruited by the community health agents who will invite mothers from the list of families assigned to them and send reminders by telephone and WhatsApp. An estimated 95% of households in Peru have access to a mobile phone (45).

Sample size calculation

Community based co-design workshops employ primarily design and qualitative methods. There is no sample size calculation based on a quantitative outcome or measures related to user and design research methods. We aim to recruit a diversity of characteristics in the health professionals (men and women, different job roles, and at different career stages/length of service in the area). Among caregivers, we aim to recruit participants with a range of characteristics relating to age, employment status, education, and number of children. We will ensure accountability and rigour through continuous "debate, critique and reflection" (46) between the participants and the research team.

Data collection methods

Prior to the participatory co-design process, the research team will create a set of design artefacts/probes and drawings to actively support the engagement of the participants, such as a visual storyboard with different scenarios (scenario-based challenge cards) reflecting the top challenges from Phase 1, and a collection of ideation/design cards that includes a deck of pictorial representations of people, resources and strategies as well as technology coming from the Phase 1 findings, research team suggestions and informed by relevant literature.

We will then conduct a series of four workshops in each site (8 in total), each one of approximately two hours duration on average including a refreshment break. The workshops will take place in parallel in the two study areas led by the same researchers to strengthen methodological consistency and continuity while also accounting for the contextual

For peer review only - http://bmjopen.bmj.com/site/about/guidelines.xhtml

differences from each study setting to adapt methods when needed. Different design ideas and co-produced artefacts can be developed in the two locations, but emerging design concepts and ideas will be cross-referenced across the co-design workshops in each site to allow for complementarity. Workshops will be audio-recorded and visual activities will be recorded by photography and workshop co-created materials. The sequence and purpose of workshops is outlined below:

i. Idea generation workshops

Community participants (e.g. mothers, family members, health facility staff and community health agents) will generate creative and innovative ideas to support healthy diets for IYC including Double Duty Actions (actions to address micronutrient deficiency and anaemia, avoiding excess intake)(8) and will discuss how to implement recommended practices (47). Methods will include using printed sketches with different scenarios and reflecting on the main challenges to healthy dietary practices identified in Phase 1. Scenarios will be read to participants who will then brainstorm using post-it notes and sketch potential solutions (supported by ideation/design cards) or interventions to assist family caregivers, government healthcare centres and staff to enhance nutrition and complementary feeding. Interventions could be digital/non-digital, targeting relevant users (i.e., households, individuals, or healthcare professionals) or the wider community or health service environment. At least three design themes will be drawn from the idea generation workshops following collective analysis and evaluation. These will be refined further e.g. using visual sketching techniques for conceptual design (48–50) and to enable future thinking (51,52). A deciding vote will select the preferred design concepts to be considered in the next workshops.

ii. Co-creating future scenarios workshops

Taking a future-oriented perspective (51), community participants will explore and co-create visions of the future/future scenarios through sketching techniques (52) and the 'futures' workshop method (53,54). First, participants will critically reflect on the design ideas/concepts/sketches that emerged from the idea generation workshops. Next, in the 'fantasy' phase, participants will divide into small groups to devise new ideas and visions of the future for further development. Use of sketching will promote collaborative reflection, innovation, and co-design of the ideal future (55). Finally, participants will discuss the envisaged concepts/scenarios and assign implementation priorities based on suitability to the

For peer review only - http://bmjopen.bmj.com/site/about/guidelines.xhtml

local communities and select up to three design concepts for further exploration as potential interventions.

iii. Storyboarding workshops

Participants will create storyboards (47) to further develop and visually convey up to three proposed design concepts. They will then elaborate and consolidate the best-case scenario for each concept. After presenting storyboards, participants will discuss the pros and the cons of each concept and a list of key functionalities that stand out for each design concept will be identified.

iv. Early implementation and feedback workshops

Early-stage implementation of the design concepts will take place through design plans, visualisations and creating material artefacts, design probes and intervention prototypes. Prototypes are proposals for future "things-that-are-not-quite-objects-yet" (56), providing insights into what might be possible in the future to current configurations of resources, people and practices. These workshops will gather early feedback on the initial design iterations through, e.g., feedback sessions and hands-on activities. At the end, up to one design concept per research site will be selected for further development based on early feedback. High-fidelity prototypes will be implemented and refined after the final workshop and before piloting.

Pilot studies to refine designed intervention prototypes

We will pilot the materials, messages and delivery methods of the intervention prototype in the health facilities or households in the study areas. This may include new approaches to staff training, novel methods for delivering messages for both health service staff or parents/caregivers and/or in the home (e.g., text messaging, games, etc.). Prototypes will be piloted for 1-2 months. Qualitative evaluation will use ethnographic methods, data logging, and pre/post questionnaires and interviews and will be adapted to the emerging characteristics of each prototype. The evaluations with community participants will explore relevance, acceptability, feasibility and impact potential of the refined version of the generated prototypes. Exit interviews with mothers attending health facilities will be used to evaluate their experiences. This project timescale is insufficient to evaluate dietary behaviour changes

For peer review only - http://bmjopen.bmj.com/site/about/guidelines.xhtml

for IYC feeding, but subsequent funding will be sought to conduct further longer-term evaluations and further redesign and refinement of the prototypes.

Data analysis

Audio-recordings of participatory workshops will be transcribed verbatim in Spanish by the same researcher who facilitates workshops. Observations, written notes, and photographs from workshops will be recorded as further supporting materials. Transcripts of workshops will be analysed along with the materials produced**,** using a collaborative tool (Miro). At each workshop, we will share the summaries and themes with participants that have come from the previous workshop, using drawings and designs to capture ideas and themes, to support discussion, reflection, and additional feedback. In depth analysis of data will take place alongside the participatory design process. Coding will be conducted in Spanish primarily by one investigator (DO) who will then check and discuss codes with co-investigators (one expert in HCI (NV) and one nutritionist (RPa)) to reach consensus. Codes will then be translated to English by one of the bilingual researchers. Qualitative data will be analysed using a combination of affinity diagramming to quickly analyse the co-produced materials to identify requirements and complemented by thematic analysis of participants discussions using NVivo software.

Ethics and dissemination

Ethical approval for this study was obtained from the Ethical Review Committee of the Instituto de Investigación Nutricional (IIN) Peru (reference 388-2019/CIEI-IIN), Loughborough University (C19-87) and confirmed by Cardiff University based on local approval in Peru. Written informed consent, and reconsent for repeat workshop participation, will be provided by all participants after receiving written and verbal information about the study. Participants will be informed of the right to withdraw from participation at any stage.

Data (audio-recordings, transcripts, drawings, prototype designs and images) will be stored on a secure workspace at Loughborough University accessible only by investigators and designated research staff during the project. During data collection, information will be stored on encrypted or password protected laptops and tablets in Peru. No personal identifying information will be associated with design outputs, and all drawings, ideas and creative output will be stored anonymously. Following the study, data will be stored on the

For peer review only - http://bmjopen.bmj.com/site/about/guidelines.xhtml

institutional research repository at Loughborough University and made open access after a period of embargo. The tools used for the survey, in-depth interviews and policy mapping have been deposited on the research repository (57–60) Data relating to Phase 1 of the project can be accessed at https://doi.org/10.17028/rd.lboro.c.6329105.v1; https://doi.org/10.17028/rd.lboro.c.6329093.v1 and https://doi.org/10.17028/rd.lboro.c.6349121.v1 Publications will follow criteria for reporting qualitative research (COREQ) guidelines (61) and guidelines for reporting patient and public involvement in research (GRIPP2) (62).

Findings of the participatory design process will be disseminated through a deliberative workshop in Lima, Peru. Community participants and policy makers will be invited to the deliberative workshop with study investigators to consider the outcomes of the pilot work. This workshop will share insights from the iterative design process and the developed prototypes. We will reflect on what worked and what did not work during the design activities, identifying factors that facilitated success (e.g., design probes (storyboard and design cards) showing great potential to facilitate community participation and ideation, active community engagement and continued participation, generation of creative and context-relevant ideas, future scenarios, and design concepts, community participants valuing and trusting the co-design process, etc) (43,63). Research findings from Phase 1 and 2 will also be disseminated through academic publications. We will seek future opportunities to continue developing the generated prototypes and rolling out the prototypes as a trial intervention with longer-term evaluation to examine the effect on complementary feeding behaviours and dietary outcomes for infant and young child nutrition.

Discussion and conclusions

The first phase of this study identified nutritional challenges relating to stunting, risk of overweight or obesity and anaemia in IYC among low income, peri-urban communities in Peru. From these identified challenges, we prioritised the top four nutritional challenges to take forward to our participatory co-design study (Phase 2). This next phase will involve healthcare professionals, community health agents and caregivers working together to iteratively co-design and test prototypes that aim to address the identified nutritional challenges. The study will offer recommendations for design, research and policy in relation to the co-design of digital health tools in the context of IYC nutrition in Peru. An important consideration in the study is that our formative research was conducted up to March 2020

For peer review only - http://bmjopen.bmj.com/site/about/guidelines.xhtml

before the COVID-19 pandemic, and our participatory-co-design phase started in June 2022 when most restrictions relating to COVID-19 were lifted. The intervening period may have influenced caregiver and healthcare professionals' ideas and perceived needs for healthy nutrition of IYC. Working across two languages, English and Spanish, also adds some complexities to the co-design process. We also recognise that the participatory co-design work conducted in Lima and Huánuco may not capture the nutritional challenges for all urban contexts in Peru, and future work would need to examine how our co-design approach and emerging prototypes could be adapted for other urban and rural environments.

For peer review only

Authors' contributions

EKR, HCK, NV, RPa, RB, MH, PG designed and led the development of the funded grant proposal. EKR, RPa, HCK, NV, RB, MH, PG, RPr, and DO developed detailed protocols for Phase 1 and the participatory co-design (Phase 2). All authors contributed to writing the manuscript and had critical input on the final version.

Contribution to public involvement

Not applicable

Funding statement

This study was supported by the UK Medical Research Council (MR/S024921/1) and CONCYTEC/ PROCIENCIA Perú (032-2019-FONDECYT) through the Newton Fund.

Competing interests' statement

The authors declare no competing interests.

Word count: 4051

Acknowledgements We gratefully acknowledge the contribution of all the investigators and field and research assistants on the PERUSANO project.

For peer review only - http://bmjopen.bmj.com/site/about/guidelines.xhtml

References

1. World Health Organization. Complementary Feeding of Young Children in Developing Countries: A Review of Current Scientific Knowledge. Geneva; 1998.

2. World Health Organization. Guiding Principles for Feeding Non-Breastfed Children 6-24 Months of Age. Geneva; 2005.

3. WHO, UNICEF, USAID, FANTA 2, UC Davis, AED. Indicators for Assessing Infant and Young Child Feeding Practices, Part 1: Definitions. Geneva; 2008.

4. Hawkes C, Harris J, Gillespie S. Changing diets: Urbanization and the nutrition transition. 2017 Global Food Policy Report. 2017;34–41.

5. Pries AM, Huffman SL, Champeny M, Adhikary I, Benjamin M, Coly AN, et al. Consumption of commercially produced snack foods and sugar-sweetened beverages during the complementary feeding period in four African and Asian urban contexts. Maternal and Child Nutrition. 2017 Oct;13:e12412.

6. Rousham EK, Goudet S, Markey O, Griffiths P, Boxer B, Carroll C, et al. Unhealthy Food and Beverage Consumption in Children and Risk of Overweight and Obesity: A Systematic Review and Meta-Analysis. Advances in nutrition (Bethesda, Md). 2022 Oct 2;13(5):1669–96.

7. Lutter CK, Grummer-Strawn L, Rogers L. Complementary feeding of infants and young children 6 to 23 months of age. Nutrition Reviews. 2021 Jul 7;79(8):825–46.

8. WHO. Double-duty actions: policy brief. Geneva; 2017.

9. Development Initiatives. 2020 Global Nutrition Report: Action on equity to end malnutrition. Bristol, UK; 2020.

10. INEI. Encuesta Demográfica y de Salud Familiar (ENDES) 2020. Lima; 2021.

11. Huicho L, Huayanay-Espinoza CA, Herrera-Perez E, Segura ER, Niño de Guzman J, Rivera-Ch M, et al. Factors behind the success story of under-five stunting in Peru: a district ecological multilevel analysis. BMC Pediatrics. 2017 Dec 19;17(1):29.

12. Ministry of Health G of P. Plan nacional para la reducción y control de la anemia materno infantil y la desnutrición crónica infantil en el Perú: 2017 – 2021. 2017.

13. INEI. Encuesta Demográfica y de Salud Familiar (ENDES) 2017. Lima; 2017.

14. Penny ME, Michelle Jimenez M, Margot Marin R. Early rapid weight gain and subsequent overweight and obesity in middle childhood in Peru. BMC Obesity. 2016 Dec 13;3(1):55.

15. Creed-Kanashiro H, Wasser HM, Bartolini R, Goya C, Bentley ME. Formative research to explore the acceptability and use of infant food grinders for the promotion of animal source foods and micronutrient powders in rural Peru. Maternal and Child Nutrition. 2018 Oct 2;14(4):e12600.

16. Holeman I, Kane D. Human-centered design for global health equity. Information Technology for Development. 2020 Jul 2;26(3):477–505.

For peer review only - http://bmjopen.bmj.com/site/about/guidelines.xhtml

17. Papoutsi C, Wherton J, Shaw S, Morrison C, Greenhalgh T. Putting the social back into sociotechnical: Case studies of co-design in digital health. Journal of the American Medical Informatics Association. 2021 Feb 15;28(2):284–93.

18. Unertl KM, Schaefbauer CL, Campbell TR, Senteio C, Siek KA, Bakken S, et al. Integrating community-based participatory research and informatics approaches to improve the engagement and health of underserved populations. Journal of the American Medical Informatics Association. 2016 Jan 1;23(1):60–73.

19. Hussain S, Sanders EBN, Steinert M. Participatory design with marginalized people in developing countries: Challenges and opportunities experienced in a field study in Cambodia. International Journal of Design. 2012;6(2):91–109.

20. Sanders EBN, Stappers PJ. Probes, toolkits and prototypes: Three approaches to making in codesigning. CoDesign. 2014 Jan 2;10(1):5–14.

21. Halskov K, Hansen NB. The diversity of participatory design research practice at PDC 2002-2012. International Journal of Human Computer Studies. 2015 Feb;74:81–92.

22. Kanstrup AM, Madsen J, Nøhr C, Bygholm A, Bertelsen P. Developments in participatory design of health information technology - A review of PDC publications from 1990 - 2016. Participatory Design & Health Information Technology. 2017;233:1–13.

23. Andía D, Charca S, Reynolds-Cuéllar P, Noel J. Community-oriented engineering co-design: case studies from the Peruvian Highlands. Humanities and Social Sciences Communications. 2022 Sep 14;9(1):311.

24. Lasage R, Muis S, Sardella CSE, van Drunen MA, Verburg PH, Aerts JCJH. A Stepwise, participatory approach to design and implement community based adaptation to drought in the Peruvian Andes. Sustainability (Switzerland). 2015 Feb 10;7(2):1742–73.

25. Smits KM, McDonald L, Smith NM, Gonzalez F, Lucena J, Martinez G, et al. Voces Mineras: Clarifying the future of artisanal and small-scale mining collaborations. Extractive Industries and Society. 2020 Jan;7(1):68–72.

26. Lazo-Porras M, Perez-Leon S, Cardenas MK, Pesantes MA, Miranda JJ, Suggs LS, et al. Lessons learned about co-creation: developing a complex intervention in rural Peru. Global Health Action. 2020 Dec 31;13(1):1754016.

27. Greene MC, Bonz A, Cristobal M, Vega C, Andersen LS, Angulo A, et al. Evaluating the feasibility of a group psychosocial intervention for migrant and host community women in Ecuador and Panamá: protocol for a multi-site feasibility cluster trial. Pilot and Feasibility Studies. 2022 Dec 15;8(1):126.

28. Braa J, Titlestad OH, Sæbø J. Participatory health information systems development in Cuba. In: Proceedings of the eighth conference on Participatory design Artful integration: interweaving media, materials and practices - PDC 04. New York, New York, USA: ACM Press; 2004. p. 53.

29. Hanington BM, Martin B. Universal Methods of Design: 125 Ways to Research Complex Problems, Develop Innovative Ideas, and Design Effective Solutions. Rockport International; 2019.

For peer review only - http://bmjopen.bmj.com/site/about/guidelines.xhtml

30. Harrington HJ. Affinity Diagrams. In: The Innovation Tools Handbook, Volume 2. Productivity Press; 2016. p. 45–54.

31. Remy C, Harboe G, Frich J, Biskjaer MM, Dalsgaard P. Challenges and Opportunities in the Design of Digital Distributed Affinity Diagramming Tools. In: European Conference on Cognitive Ergonomics 2021. New York, NY, USA: ACM; 2021. p. 1–5.

32. Holtzblatt K, Beyer H. Contextual design: defining customer-centered systems. Elsevier; 1997.

33. Harboe G, Huang EM. Real-World Affinity Diagramming Practices. In: Proceedings of the 33rd Annual ACM Conference on Human Factors in Computing Systems. New York, NY, USA: ACM; 2015. p. 95–104.

34. Carroll M, Goldman S, Britos L, Koh J, Royalty A, Hornstein M. Destination, Imagination and the Fires Within: Design Thinking in a Middle School Classroom. International Journal of Art & Design Education. 2010 Mar 15;29(1):37–53.

35. Chatty T, Qu Y, Ba-Sabaa HH, Murnane EL. Examining the user experience of life cycle assessment tools and their ability to cater to ecodesign in early-stage product development practice. Proceedings of the Design Society. 2021 Aug 27;1:1441–50.

36. Kim S, Jung DHS, Upender A, Claire S, Esfandiari I. Human-Food Interaction Framework: A New Design Tool Used to Understand Amateur Home Cooks' Needs. In: International Conference on Human-Computer Interaction. Springer; 2019. p. 9–14.

37. Bødker S, Ehn P, Knudsen J, Kyng M, Madsen K. Computer support for cooperative design (invited paper). In: Proceedings of the 1988 ACM conference on Computer-supported cooperative work - CSCW '88. New York, New York, USA: ACM Press; 1988. p. 377–94.

38. Bϕdker S, Grϕnbæk K, Kyng M. Cooperative design: techniques and experiences from the Scandinavian scene. In: Readings in Human–Computer Interaction. Morgan Kaufmann; 1995. p. 215–24.

39. Bødker S. When second wave HCI meets third wave challenges. In: ACM International Conference Proceeding Series. New York, New York, USA: ACM Press; 2006. p. 1–8.

40. Clemensen J, Larsen SB, Kyng M, Kirkevold M. Participatory design in health sciences: Using cooperative experimental methods in developing health services and computer technology. Qualitative Health Research. 2007 Jan 1;17(1):122–30.

41. Akoglu C, Dankl K. Co-creation for empathy and mutual learning: a framework for design in health and social care. CoDesign. 2021 Jul 3;17(3):296–312.

42. INEI. Encuesta Demográfica y de Salud Familiar (ENDES) 2019. Lima; 2020.

43. Bird M, McGillion M, Chambers EM, Dix J, Fajardo CJ, Gilmour M, et al. A generative co-design framework for healthcare innovation: development and application of an end-user engagement framework. Res Involv Engagem. 2021 Dec 1;7(1):12.

44. Till S, Farao J, Coleman TL, Shandu LD, Khuzwayo N, Muthelo L, et al. Community-based Co-design across Geographic Locations and Cultures: Methodological Lessons from Co-design Workshops in South Africa. In: Participatory Design Conference 2022: Volume 1. New York, NY, USA: ACM; 2022. p. 120–32.

For peer review only - http://bmjopen.bmj.com/site/about/guidelines.xhtml

45. INEI. Evolución de la Pobreza Monetaria 2009–2020: Encuesta Nacional de Hogares, 2010–2020 (Informe Técnico). Lima; 2020.

46. Frauenberger C, Good J, Fitzpatrick G, Iversen OS. In pursuit of rigour and accountability in participatory design. International Journal of Human Computer Studies. 2015 Feb;74:93–106.

47. Erickson T. Design as Storytelling. In: Interactions. ACM Publications; 1996. p. 30–5.

48. McGown A, Green G, Rodgers PA. Visible ideas: Information patterns of conceptual sketch activity. Design Studies. 1998;19(4):431–53.

49. Rodgers PA, Green G, McGown A. Using concept sketches to track design progress. Design Studies. 2000;21(5):451–64.

50. Chen S. The Design Studio Approach. In: The Design Imperative. Champaign, Illinois: Palgrave Macmillan; 2019. p. 55–70.

51. Kjærsgaard MG, Halse J, Smith RC, Vangkilde KT, Binder T, Otto T. Introduction: design anthropological futures. In: Design Anthropological Futures. Routledge; 2020. p. 1–16.

52. Sturdee M, Lindley J. Sketching & drawing as future inquiry in HCI. ACM International Conference Proceeding Series. 2019;1–10.

53. Kensing F, Madsen H. Generating Visions: Future Workshops and Metaphorical. In: Design at Work: Cooperative Design of Comuter Systems. CRC Press; 1991.

54. Jungk K, Müllert N. No Title. In: Future Workshops: How to create desirable futures. Institute for Social Inventions; 1987.

55. Sanders EBN, Stappers PJ. Co-creation and the new landscapes of design. CoDesign. 2008 Mar;4(1):5–18.

56. Corsín Jiménez A. Introduction: The prototype: more than many and less than one. Journal of Cultural Economy. 2014 Oct 2;7(4):381–98.

57. Rousham E, Pradeilles R, Pareja R, Creed-Kanashiro HM. The PERUSANO study: questionnaire for quantitative survey [Internet]. Loughborough University; 2022 [cited 2023 Oct 31]. Available from: https://doi.org/10.17028/rd.lboro.18750458

58. Rousham E, Pradeilles R, Pareja R, Creed-Kanashiro H, Bartolini R. The PERUSANO study: Caregivers, health centre and day care centre influences on infant and young child feeding practices: interview guides for primary caregivers [Internet]. Loughborough University; 2022 [cited 2023 Oct 31]. Available from: https://doi.org/10.17028/rd.lboro.21711332.v2

59. Rousham E, Pradeilles R, Pareja R, Creed-Kanashiro H, Bartolini R. The PERUSANO study: Caregivers, health centre and day care centre influences on infant and young child feeding practices: interview guides for health facilities [Internet]. Loughborough University; 2022 [cited 2023 Oct 31]. Available from: https://doi.org/10.17028/rd.lboro.21717248

60. Rousham ERV, Pradeilles R, Pareja R, Creed-Kanashiro H, Holdsworth M, Rojas V. Policy mapping of double-duty actions to address multiple forms of malnutrition among infants and young children in Peru: data collection tools [Internet]. Loughborough University; 2022 [cited 2023 Oct 31]. Available from: https://doi.org/10.17028/rd.lboro.21739679.v1

For peer review only - http://bmjopen.bmj.com/site/about/guidelines.xhtml

61. Tong A, Sainsbury P, Craig J. Consolidated criteria for reporting qualitative research (COREQ): A 32-item checklist for interviews and focus groups. International Journal for Quality in Health Care. 2007 Sep 16;19(6):349–57.

62. Staniszewska S, Brett J, Simera I, Seers K, Mockford C, Goodlad S, et al. GRIPP2 reporting checklists: Tools to improve reporting of patient and public involvement in research. BMJ (Online). 2017 Aug 2;358:j3453.

63. Manafo E, Petermann L, Mason-Lai P, Vandall-Walker V. Patient engagement in Canada: a scoping review of the 'how' and 'what' of patient engagement in health research. Health Res Policy Syst. 2018 Dec 7;16(1):5.

For peer review only

For peer review only - http://bmjopen.bmj.com/site/about/guidelines.xhtml

Table 1. Top ten opportunities for intervention in infant and young child (IYC) nutrition identified and prioritised through formative research and scoping workshops to take forward to the participatory design phase

**Opportunity statements identified and prioritised by the research team†**
Reduce the consumption of unhealthy foods and beverages amongst mothers and IYC (6-23 months) to improve the overall quality of diet/feeding practices
Develop strategies to reduce inequalities to protect vulnerable populations in relation to the double burden of malnutrition
Improve emotional and psychological wellbeing for mothers to reduce its impact on IYC nutrition
Increase/enhance maternal nutrition knowledge on appropriate feeding practices (e.g., iron supplementation, complementary feeding, breastfeeding)
Help to implement governmental policies for early childhood education services to provide and promote healthy food choices
Increase adherence to iron supplementation for IYC
Enhance the uptake, access, and intercultural approaches to improve maternal and child care services
Enhance counselling skills for healthcare professionals
Improve nutrition counselling during pregnancy and from birth onwards
Address the challenges in coordinating the implementation and evaluation of policy across different levels of governmental institutions in relation to the double burden of malnutrition

†The full list of opportunities which were voted on to prioritise the 10 ten opportunities is provided in Supplementary Table 1.

For peer review only - http://bmjopen.bmj.com/site/about/guidelines.xhtml

Figure legends

Figure 1: Overview of the PERUSANO study; Phase 1 summarises the formative research objectives and Phase 2 outlines the protocol for the participatory co-design

Figure footnote: FFQ= food frequency questionnaire; IYC= infants and young children

For peer review only

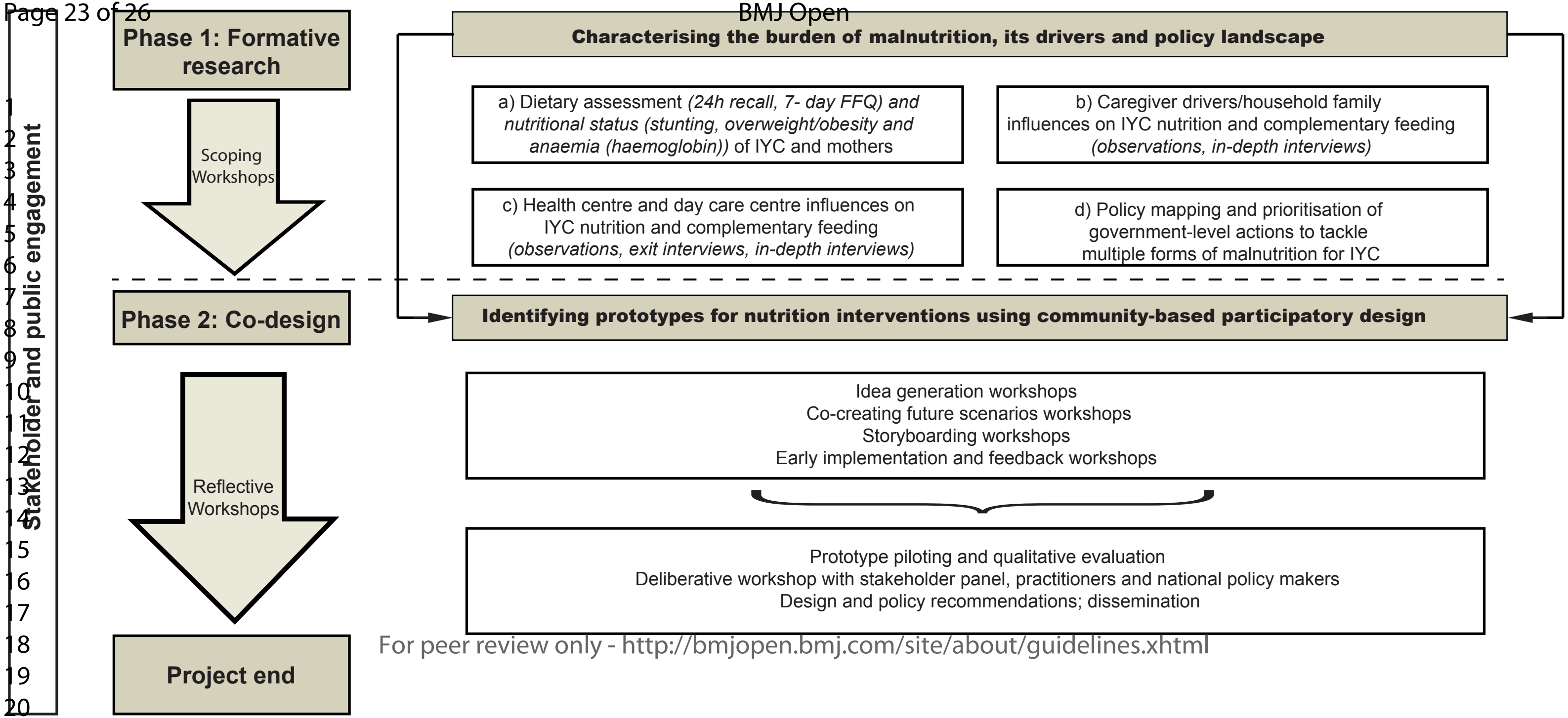

For peer review only - http://bmjopen.bmj.com/site/about/guidelines.xhtml

Designing intervention prototypes to improve infant and young child nutrition in Peru: A participatory design study protocol

E. K. Rousham, R. Pareja, H.M. Creed-Kanashiro, R. Bartolini, R. Pradeilles, D. Ortega-Roman, M. Holdsworth, P. Griffiths, N. Verdezoto

Supplementary materials

Supplementary Table 1: Summary of the 27 identified nutrition challenges, and 19 opportunities identified for infants and young children (IYC) aged 6-23 months in two peri-urban communities in Peru prior to the participatory co-design phase of the study

Overarching theme	Challenges identified in the formative research‡	Opportunity statements derived from the challenges
Improve the healthiness of maternal diets and infant & young child feeding practices	1. High consumption of sugar-sweetened beverages in mothers and IYC	1. Reduce the consumption of unhealthy foods and beverages amongst mothers and IYC to improve the overall quality of diet and feeding practices
	2. High consumption of unhealthy foods (savoury snacks/fried foods, sweet products) in mothers and IYC	
	3. Sub-optimal dietary diversity, particularly around animal-source foods and fruit and vegetable consumption, due to small quantities consumed	2. Improve the diversity of diets for mothers and IYC
	4. Inadequate engagement with responsive feeding in the home	3. Increase the engagement with responsive feeding practices at home
	5. Low prevalence of exclusive breastfeeding under six months, including early introduction of complementary foods†	4. Increase the prevalence of exclusive breastfeeding for infants under 6 months
	6. Low compliance with taking iron supplements in IYC†	5. Increase adherence to iron supplementation
	7. Issues with emotional and psychological wellbeing of mothers which impacts on the caring and feeding practices for the child	6. Improve emotional and psychological wellbeing for mothers to reduce its impact on IYC nutrition
	8. Mothers' understanding of nutrition knowledge and perception and practices around infant nutrition	7. Increase/enhance maternal nutrition knowledge on appropriate feeding practices (e.g., iron supplementation, complementary feeding, breastfeeding)
	6. Low compliance with taking iron supplements in IYC†	
	5. Low prevalence of exclusive breastfeeding under 6 months (which means that other liquids and complementary foods are introduced early)†	
	9. Challenges with regards to maternal time use in the home and outside the home	8. Improve the maternal care experience and practices at home/in everyday life -- e.g., tensions with work and time allocation
Enhance the local systems in the community to improve maternal and child nutrition	10. Lack of physical resources and materials	9. Enhance the physical and/or material resources within healthcare settings
	11. Issues with the organisation of consultations, including health centre structures and workflow	10. Optimise the organisation of consultations and workflow within healthcare settings
	12. Health service recommendations do not take into account the socio-cultural practices and norms of caregivers	11. Enhance the uptake, access, and intercultural approaches to improve maternal and child care services

For peer review only - http://bmjopen.bmj.com/site/about/guidelines.xhtml

	13. Issues with the uptake and access to health services for IYC†	
	14. Issues with regards to counselling approaches and staff skills within health centres	12. Enhance counselling skills for healthcare professionals
	15. Community-based activities for breastfeeding outreach and support have national coverage	13. Facilitate community-based activities to enhance breastfeeding practices
	16. Inadequate counselling for complementary feeding	14. Improve nutrition counselling during pregnancy and from birth onwards
	17. Inadequate nutritional counselling for pregnant women	
	18. Restriction on infant formula promotion in ante-natal and post-natal settings	15. Promote the reduction of the use and promotion infant formula in antenatal and postnatal care settings
	19. Government policy should ensure food services in early childhood education provide and promote healthy food choices (e.g. via canteens, food at events, fundraising, promotions, vending machines)†	16. Help to implement governmental policies for early childhood education services to provide and promote healthy food choices
Support the national system to improve maternal and child nutrition policy implementation	20. Funding for interventions and policies to reduce obesity, stunting and iron deficiency anaemia in IYC	17. Seek funding opportunities to develop interventions and policies to address the double burden of malnutrition
	21. Monitoring systems particularly in vulnerable young children's populations for the five double duty actions and to track progress towards reducing nutritional inequalities	18. Develop strategies to reduce inequalities to protect vulnerable populations in relation to the double burden of malnutrition
	22. Reduce inequalities or protect vulnerable populations in relation to obesity, stunting and iron deficiency anaemia in IYC	
	23. Lack of use of ICT interventions for the double burden of malnutrition in low and middle income country settings*	
	13. Issues with the uptake and access to health services for IYC†	
	24. Restrict commercial influences on the development of policies related to preventing obesity, stunting and iron deficiency anaemia in IYC	
	25. Robust coordination to ensure alignment, and integration of policies in obesity, stunting and iron deficiency anaemia in IYC across departments and levels of government (national and local)	19. Address the challenges in coordinating the implementation and evaluation of policy across different levels of governmental institutions in relation to the double burden of malnutrition
	26. Lack of coherence in the policy environment (e.g. lack of a holistic nutrition approach, no attention to the double burden of malnutrition	
	19. Government policy should ensure food services in early childhood education provide and promote healthy food choices (e.g. via canteens, food at events, fundraising, promotions, vending machines)†	

For peer review only - http://bmjopen.bmj.com/site/about/guidelines.xhtml

	27. Low adherence to taking iron supplements, reasons include fear of side effects and difficulties of administering at home, and low quality iron supplements that government offers	

‡Shading represents the source of each challenge from Phase 1 work packages. Key: no fill = work package a) Dietary assessment and nutritional status of IYC and mothers; light grey = work package b) Caregiver drivers and household or family influences on IYC nutrition and complementary feeding; mid-grey = work package c) Health centre and day care centre influences on IYC nutrition and complementary feeding; dark-grey = work package d) Policy mapping and prioritisation of government-level actions to tackle multiple forms of malnutrition for IYC.

†Challenges that appeared under more than one overarching theme

*Challenge identified from reviewing literature

For peer review only
